# Direct C−H Trifluoromethylation of (Hetero)Arenes in Water Enabled by Organic Photoredox‐Active Amphiphilic Nanoparticles

**DOI:** 10.1002/chem.202201322

**Published:** 2022-07-28

**Authors:** Fabian Eisenreich, Anja R. A. Palmans

**Affiliations:** ^1^ Laboratory of Macromolecular and Organic Chemistry Institute of Complex Molecular Systems Department of Chemical Engineering and Chemistry Eindhoven University of Technology P.O. Box 513 5600 MB Eindhoven (The Netherlands

**Keywords:** enzyme mimics, green chemistry, nanoparticles, photoredox catalysis, trifluoromethylation

## Abstract

Photoredox‐catalyzed chemical conversions are predominantly operated in organic media to ensure good compatibility between substrates and catalysts. Yet, when conducted in aqueous media, they are an attractive, mild, and green way to introduce functional groups into organic molecules. We here show that trifluoromethyl groups can be readily installed into a broad range of organic compounds by using water as the reaction medium and light as the energy source. To bypass solubility obstacles, we developed robust water‐soluble polymeric nanoparticles that accommodate reagents and photocatalysts within their hydrophobic interior under high local concentrations. By taking advantage of the high excited state reduction potential of *N*‐phenylphenothiazine (PTH) through UV light illumination, the direct C−H trifluoromethylation of a wide array of small organic molecules is achieved selectively with high substrate conversion. Key to our approach is slowing down the production of CF_3_ radicals during the chemical process by reducing the catalyst loading as well as the light intensity, thereby improving effectiveness and selectivity of this aqueous photocatalytic method. Furthermore, the catalyst system shows excellent recyclability and can be fueled by sunlight. The method we propose here is versatile, widely applicable, energy efficient, and attractive for late‐stage introduction of trifluoromethyl groups into biologically active molecules.

## Graphical abstract

**Photoredox‐catalyzed** trifluoromethylations of a variety of small organic molecules were studied in water with the aid of amphiphilic polymeric nanoparticles. Excellent recyclability of the nanoparticles, high efficacy and selectivity, and the use of low intensity LED or sunlight render this method versatile and environmentally friendly.

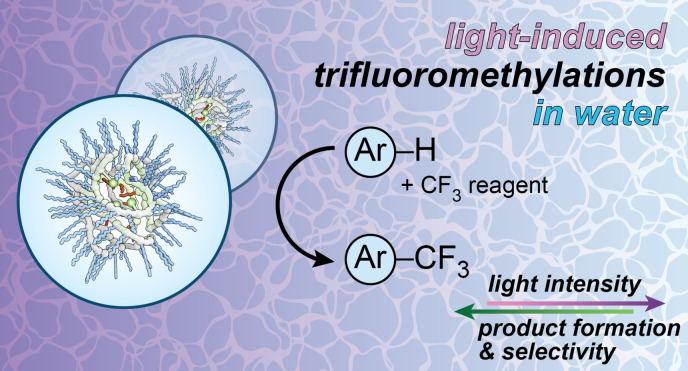

## Introduction

Reduction of waste and energy consumption as well as utilizing renewable feedstock materials and environmentally benign substances are cornerstones of sustainable chemical processes.[^1^, ^2^, ^3^] As a consequence, green chemistry principles are increasingly implemented by chemists when developing modern synthetic methods.[^4^, ^5^] Catalysis plays an integral role in achieving the objectives of sustainable chemistry.[^6^, ^7^] Recently, the extensive progress in photoredox catalysis has facilitated a large number of demanding chemical transformations that are unattainable by traditional thermal catalysis.[^8^, ^9^, ^10^, ^11^, ^12^, ^13^, ^14^, ^15^, ^16^] In photoredox catalysis, reactive radicals of low‐energy organic molecules are generated via single‐electron‐transfer processes with the excited photocatalysts. Using the energy of (sun)light instead of thermal activation to break and create stable chemical bonds is an auspicious green approach.^[17]^

Surprisingly, despite these considerable achievements, the performance of photoredox catalysis in aqueous solutions is still in its infancy.[^18^, ^19^] Yet, using water as an alternate reaction medium has multiple advantages.[^20^, ^21^, ^22^] Water is abundant, inexpensive, non‐toxic, biocompatible, and by far the most environmentally benign solvent. Additionally, the solubility of molecular oxygen in water is comparably low,^[23]^ a property that is crucial for oxygen‐sensitive (photocatalytic) processes. However, the immiscibility of water and many photocatalysts, reactants, or other additives remains a major hurdle to take when efficient photocatalytic processes are desired in water.

In order to shift photoredox catalysis to aqueous solutions, various strategies have been developed. For example, rendering the reaction partners water‐soluble is an interesting approach to ensure good compatibility between substrates and catalysts. The Xue group conducted the arylation of pyridines with aryldiazonium salts in pure water by the use of a water‐soluble ruthenium(II) polypyridyl complex.^[24]^ Other examples of aqueous photoredox catalysis are cyclization reactions to construct building blocks such as dihydroisoquinolinones,^[25]^ benzo[*a*]fluoren‐5‐ones,^[26]^ oxindoles,^[27]^ and thioflavones.^[28]^

In addition, encapsulation of water‐insoluble components within hydrophobic compartments has been evaluated,^[29]^ which has the advantage that it can be applied to virtually any water‐compatible organic reaction. The reactants thereby gather under high local concentrations, which can speed up chemical reactions[^30^, ^31^] and lead to exceptional selectivities.[^32^, ^33^] Lipshutz et al. reported on the photoreductive sulfonylation of alkenes by the use of an amphiphilic photocatalyst composed of an iridium complex covalently bound to a micellar reagent.^[34]^ Recently, the König group observed that the stability of Ir(dtbby)(ppy)_2_PF_6_ in its excited state increased in the presence of sodium lauryl ether sulfate as the micelle‐forming reagent in water.^[35]^ This boost in stability was exploited to transform non‐activated alkyl chlorides in dehalogenation, addition, and cyclization reactions.

Instead of using micelles to sequestrate water‐insoluble reagents, amphiphilic polymers can also be applied as modular platform to conduct light‐induced reduction and C−C cross‐coupling reactions in aqueous medium.[^36^, ^37^] Due to the hydrophobic effect, these macromolecules collapse in water into single‐chain polymeric nanoparticles (SCPNs).[^38^, ^39^, ^40^, ^41^, ^42^] In contrast to micellar systems, SCPNs do not have a critical aggregation concentration and act as unimolecular micelles that function as nanoreactors up to very high dilution.[^38^, ^39^] SCPNs are typically about 10 nm in size, provide a robust and hydrophobic interior, and can conveniently be modified and recycled.[^36^, ^38^] SCPNs have successfully been used in a variety of aqueous reactions such as Ru‐catalyzed oxidations/reductions,^[32]^ proline‐catalyzed aldol reactions,^[38]^ and Cu‐catalyzed click reactions.[^43^, ^44^]

Since 18 out of the top 200 best‐selling small molecule pharmaceuticals contain trifluoromethyl groups,^[45]^ late‐stage modifications under mild conditions are highly sought‐after.[^46^, ^47^, ^48^, ^49^, ^50^, ^51^, ^52^, ^53^] Although significant progress regarding aqueous trifluoromethylation reactions has been made,[^54^, ^55^] there are currently only a few photocatalytic methods that operate in water.[^25^, ^56^, ^57^] These protocols are restricted in their substrate scope,[^25^, ^56^, ^57^] use rare ruthenium or iridium‐based catalysts,[^25^, ^57^] require long reaction times of up to two days,^[56]^ or apply irradiation equipment with high energy consumption (e. g., photoreactors).^[57]^

**Figure 1:**
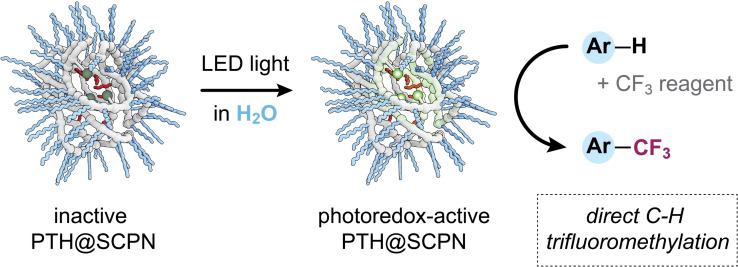
Schematic representation of the direct C−H trifluoromethylation of (hetero)arenes by photoredox catalyst (PTH) containing SCPNs in water.

Hence, a broadly applicable, catalytically and energetically efficient, light‐driven derivatization of organic compounds with CF_3_ groups in aqueous solutions remains experimentally unattained. Here, we develop a photocatalytic, metal‐free method for the direct C−H trifluoromethylation of a wide array of (hetero)arenes without the necessity of pre‐functionalization in pure water and at room temperature (Figure 1). As catalytic system we use SCPNs decorated with different ratios of *N*‐phenylphenothiazine moieties, which act as highly reductive organic photoredox catalysts under illumination with operationally simple and commercially available light‐emitting diodes (LEDs) run at very low light intensity.

## Results and Discussion

### Design, synthesis, and characterization of photoredox‐active SCPNs

**Figure 2:**
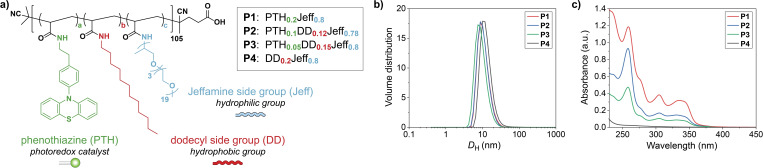
Design and characterization of amphiphilic catalytically active SCPNs. a) Chemical structure of polymers **P1**–**P4**, b) DLS measurements of **P1**–**P4** in water (c=1.0 mg/mL, T=20 °C), c) UV/vis absorption spectra of **P1**–**P4** in water (c=0.3 mg/mL, T=20 °C).

For our study, we designed four differently substituted amphiphilic polymers (**P1**–**P4**) to screen for optimal catalytic activity (Figure 2a). The polymeric backbone of these macromolecules was uniformly decorated with long polyetheramine‐based chains (ca. 80 %), so‐called Jeffamine®M‐1000, to bestow water‐solubility. Yet, the polymers differ in the ratio of catalytically active pendants and hydrophobic side chains. We selected *N*‐phenylphenothiazine as a robust metal‐free photoredox catalyst, which is simple to modify and known to facilitate various chemical transformations.[^58^, ^59^, ^60^, ^61^] To increase the hydrophobic content that furnishes the interior of SCPNs, we introduced linear dodecyl side groups. While **P1** is solely functionalized with PTH units (20 %), **P2** and **P3** are loaded with a lower amount of catalyst (10 % and 5 %, respectively). To set the overall hydrophobic content of **P2** and **P3** to 20 %, they are additionally modified with dodecyl groups. Furthermore, we prepared **P4**, which only contains dodecyl side groups as the hydrophobic part. As **P4** is catalytically inactive, it serves as a control compound. In addition, **P4** allows to encapsulate non‐bound PTH catalysts within its hydrophobic compartments to examine the effect of covalently linking the catalytic unit to the polymeric scaffold.

In order to synthesize **P1**–**P4**, we chose a post‐functionalization approach of poly(pentafluorophenyl acrylate), which has the advantage that the same polymeric precursor is used and thus variations in chain length or molar mass dispersity are avoided.^[40]^ First, poly(pentafluorophenyl acrylate) with an average degree of polymerization of 105 and a molar mass dispersity of *Đ*=1.12 was prepared by RAFT polymerization. *Đ* was determined by size exclusion chromatography (SEC) in THF calibrated with polystyrene standards (see Figure S1 in the Supporting Information). The introduction of side groups was achieved in a straightforward fashion by sequentially adding amine‐bearing functional groups to the activated ester containing polyacrylate in THF at 50 °C (see Figures S2–S5 in the Supporting Information). To this end, the chemical structure of PTH was slightly altered by tying an ethylamine linker to the 4‐position of the phenyl group. The degree of functionalization after each addition step was conveniently determined by comparing the signals of released pentafluorophenol and remaining polymer in ^19^F NMR spectroscopy. After dialysis, the set of amphiphilic polymers **P1**–**P4** was fully characterized. The polymers’ molar mass dispersities *Đ* range between 1.19–1.28 (SEC in DMF with LiBr, Figure S6) and 1.29–1.32 (SEC in PBS, Figure S7). Dynamic light scattering (DLS) measurements showed that the average hydrodynamic diameters *D*_H_ of the polymeric nanoparticles in water lie between 10–13 nm (Figure 2b). These values are in line with previous reports on polymer strands that occupy single‐folded conformations in solution.[^36^, ^38^, ^40^, ^62^, ^63^] Thus, the formation of SCPNs in aqueous medium appears to be unaffected by the composition of the hydrophobic content (i. e., aliphatic and aromatic side groups). The optical properties of **P1**–**P4** were analyzed by UV/vis spectroscopy in water (Figure 2c), which shows the characteristic absorption profile of the aromatic catalytic unit (λ_abs_≤400 nm). The successful incorporation of the PTH photocatalyst was further confirmed by ^1^H NMR spectroscopy (see Figures S8–S11 in the Supporting Information).

### Optimizing the direct C‐H trifluoromethylation of *N*‐phenylpyrrole

**Figure 3:**
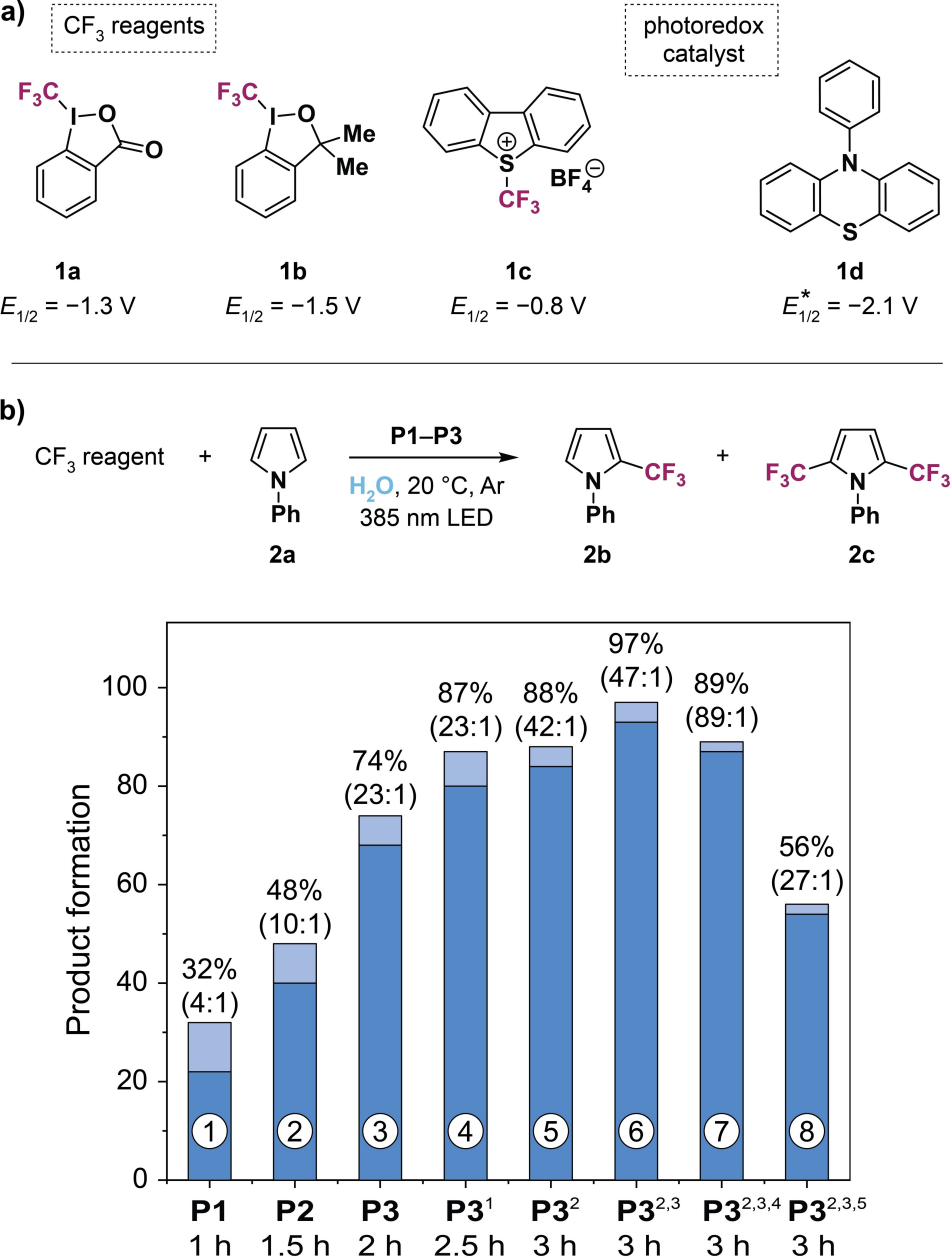
Screening for optimal reaction conditions. a) Chemical structures and reduction potentials of trifluoromethylation reagents **1 a**–**1 c** and *N*‐phenylphenothiazine catalyst **1 d**. b) Direct C−H trifluoromethylation of *N*‐phenylpyrrole **2 a** as a model substrate employing the following reaction conditions: 30 μmol of **1 a** and **2 a** as well as 10 mg of polymer were taken up in 0.8 mL of deionized water, degassed with argon, and illuminated with a 385 nm LED (*E*=92 mW/cm^2^) for the indicated time. Product formation was determined by quantitative ^19^F NMR spectroscopy using trifluorotoluene as internal standard and is specified as a sum of **2 b** (dark blue bars) and **2 c** (light blue bars) with respect to **1 a**. Molar ratios between **2 b** and **2 c** are given in parentheses. Deviations from reaction conditions: ^1^ *E*=46 mW/cm^2^, ^2^ *E*=23 mW/cm^2^, ^3^45 μmol of **2 a** (1.5 equiv), ^4^Togni's reagent **1 b** was used, ^5^Umemoto's reagent **1 c** was used.

After successfully synthesizing and characterizing **P1**–**P4**, we investigated the photocatalytic activity of these amphiphilic nanoparticles towards the direct C−H trifluoromethylation of aromatic compounds in pure water. As potential CF_3_ sources we selected Togni's reagents **1 a** and **1 b** as well as Umemoto's reagent **1 c** (Figure 3a). The one‐electron reduction potentials $E_{1/2}^{{}^{*}}$ of these reagents range between −0.8 and −1.5 V vs. standard calomel electrode (SCE).^[64]^ Since these values are above the excited state reduction potential of PTH **1 d** ($E_{1/2}^{{}^{*}}$ =−2.1 V vs. SCE),^[58]^ a single‐electron transfer from the excited photocatalyst to the CF_3_ reagent and consequently the generation of reactive CF_3_ radicals are expected.

First, we screened for optimal reaction conditions regarding the trifluoromethylation of *N*‐phenylpyrrole **2 a** as a model substrate with CF_3_ reagent **1 a** under UV light irradiation (λ_LED_=385 nm). To this end, we dissolved 10 mg of SCPN in 0.8 mL of deionized water, added equal molar amounts of **1 a** and **2 a**, and degassed the aqueous mixture with argon. After adding the hydrophobic substrates, the homogenous SCPN solution turned into a heterogenous mixture (see Figure S12 in the Supporting Information). The reaction mixture was illuminated until (near) full conversion of the CF_3_ reagent was reached and product formation was determined by quantitative ^19^F NMR spectroscopy with trifluorotoluene as internal standard. Employing polymer **P1** (catalyst loading of 7 mol % under the applied conditions) showed the generation of singly trifluoromethylated species **2 b** as the main product (22 % with respect to **1 a**) and bistrifluoromethylated compound **2 c** as the minor product (10 %) with a molar ratio of 4 : 1 (see bar 1 in Figure 3b).

Interestingly, product formation and selectivity increased with less photocatalyst attached to the SCPNs by using **P2** and **P3** with 4 and 2 mol % catalyst loadings, respectively (Figure 3b, bars 2 and 3). From these results it is apparent that a rapid production of reactive CF_3_ radicals is caused under the applied reaction conditions. With a higher amount of catalyst located within the SCPNs the formation of bistrifluoromethylated product **2 c** is more pronounced due to the presumably high local concentration of radicals. Additionally, side reactions are prone to occur, such as the generation of trifluoromethane (see Figure S13 in the Supporting Information), thereby diminishing the product formation.

In order to minimize the production of bistrifluoromethylated species **2 c** and other side products, we slowed down the CF_3_ radical production. We realized this by reducing the number of emitted photons from the LED. Indeed, dimming the UV light irradiance *E* from 92 to 46 and 23 mW/cm^2^ significantly enhanced product formation and selectivity (Figure 3b, bars 4 and 5). This crucial optimization step accentuates the significance of light intensity in photocatalytic processes. Ultimately, near quantitative product formation of 97 % with a molar ratio **2 b**/**2 c** of 47 : 1 was achieved by slightly increasing the quantity of pyrrole **2 a** to 1.5 equivalents, which led to a more effective trapping of the generated CF_3_ radicals (Figure 3b, bar 6).

Furthermore, we applied the optimized reaction conditions to CF_3_ sources **1 b** and **1 c**. Togni's reagent **1 b** showed a slightly lower product formation of 89 % but an improved selectivity (molar ratio **2 b**/**2 c**=89 : 1, Figure 3b, bar 7). Cleaving the CF_3_ group from Umemoto's reagent **1 c** resulted in the competing trifluoromethylation of released dibenzothiophene (ca. 22 %, see Figure S14) and thus less of the desired products was obtained (Figure 3b, bar 8).

### Examining the substrate scope

**Figure 4:**
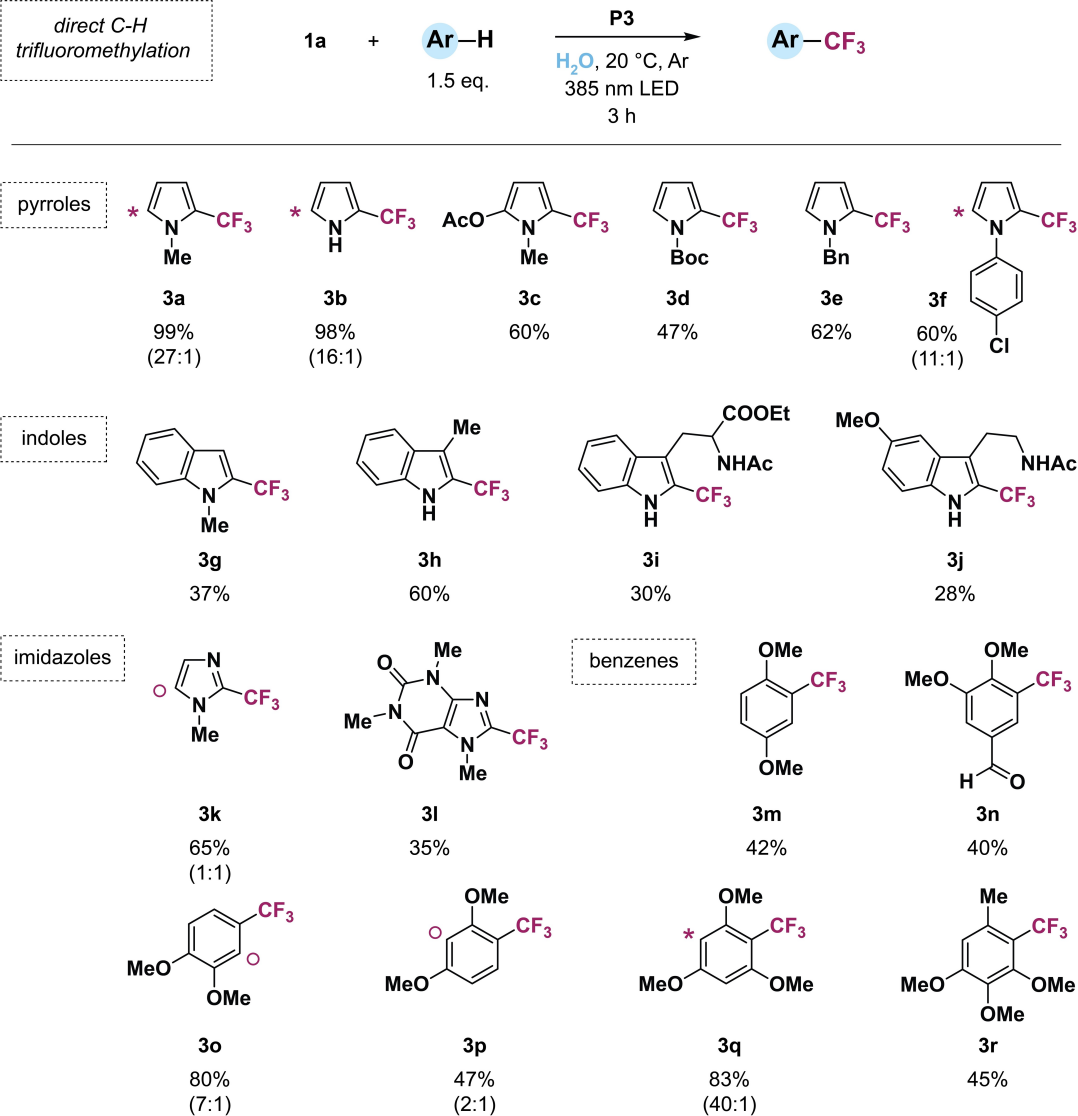
Direct C−H trifluoromethylation of various (hetero)aromatic substrates under optimized reaction conditions in water. Product formation was determined by quantitative ^19^F NMR spectroscopy using trifluorotoluene as internal standard. Product formation includes bistrifluoromethylated products or regioisomers if applicable. Position of second CF_3_ group in minor bistrifluoromethylated products is labeled with (*) and position of CF_3_ group in minor regioisomers is marked with (**○**). Molar ratios of major and minor products are given in parentheses.

To examine the applicability of our light‐driven aqueous trifluoromethylation protocol, we employed an array of (hetero)arene substrates (Figure 4). Based on the optimization results, we chose to use SCPN **P3** with the lowest PTH catalyst loading, Togni's reagent **1 a**, and an illumination time for 3 h with dimmed 385 nm light (*E*=23 mW/cm^2^). First, we tested various pyrrole derivatives which are one of the most explored heterocycles in drug discovery programs.^[65]^ Similarly to **2 a**, quantitative product formation was obtained for **3 a** and **3 b**, including the formation of bistrifluoromethylated products. High tolerance to functional groups, such as acetyl, Boc, and benzyl groups, was demonstrated with the clean conversion to products **3 c**–**3 e**.

The cleavage of aromatic carbon halogen bonds under photoreductive conditions with PTH[^58^, ^59^] and other catalysts^[66]^ in organic solvents is well established. However, under these mild conditions, aromatic C−Cl bonds remain intact, as shown with product **3 f**, providing the opportunity for consecutive functionalization (e. g., cross‐coupling reactions). We further converted indoles **3 g**–**3 j** and imidazoles **3 k** and **3 l** as other *N*‐heterocyclic building blocks to their trifluoromethylated counterparts, including biologically active compounds (amino acid tryptophan **3 i**, hormone melatonine **3 j**, and stimulant caffeine **3 l**). Lastly, various electron‐rich benzene derivatives **3 m**–**3 r** were subjected to our aqueous trifluoromethylation method and successfully transformed in medium to good yields. Diminished product formations originate from inefficient trapping of the CF_3_ radicals with the substrates thus leading to undesired side reactions (e. g., generation of trifluoromethane). Notably, all of the tested substrates are not or poorly water‐soluble in the low volume of water, which emphasizes the importance of providing hydrophobic space within the SCPNs for the photocatalytic process to occur.

### Mechanism investigation and recycling study

**Figure 5:**
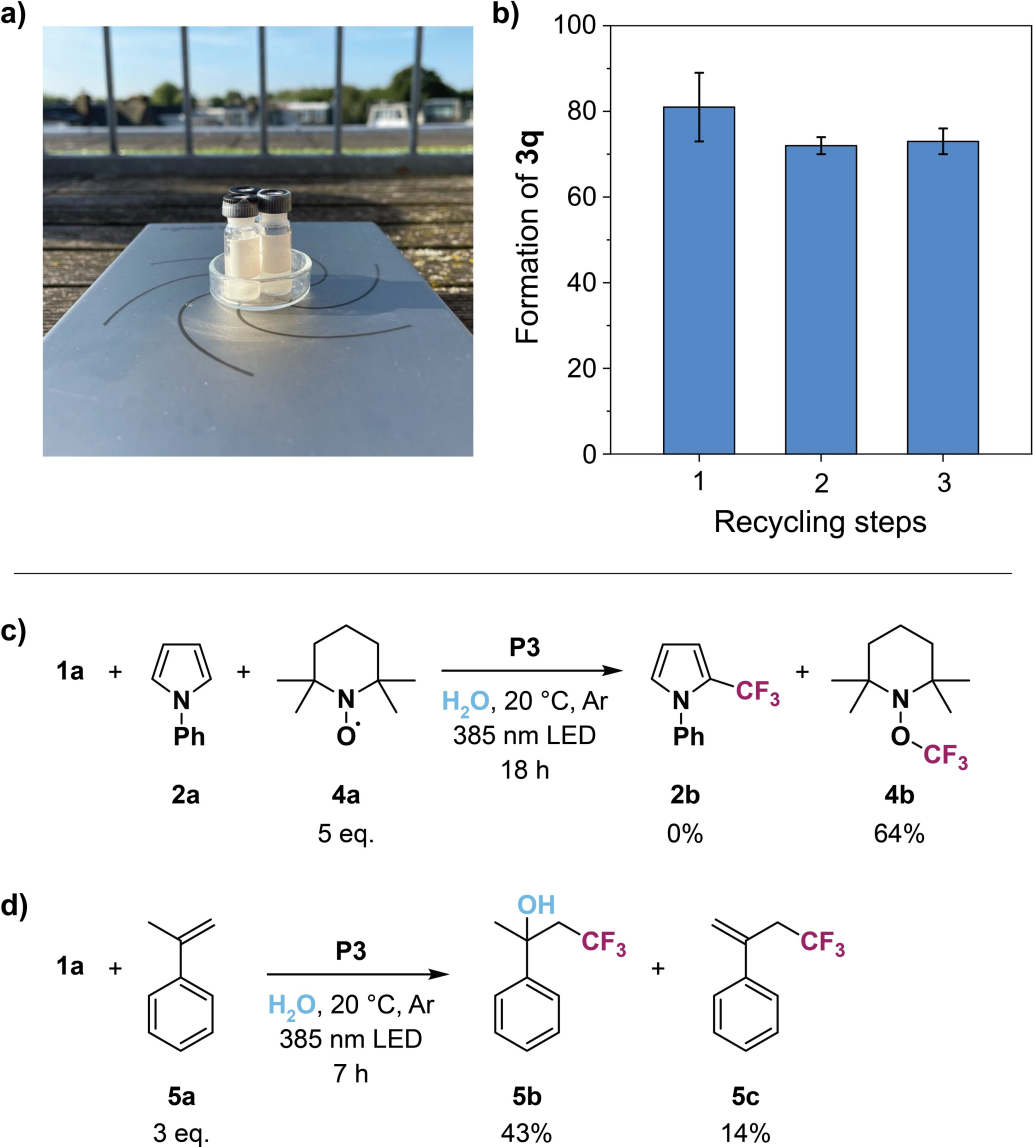
Experiments demonstrating the versatility of the photoredox‐active SCPNs: a) Photograph of the setup for the trifluoromethylation of **2 a** using optimized conditions under sunlight. b) Recycling experiments based on the consecutive synthesis of **3 q** using optimized conditions (experiment performed in duplicate). Mechanistic investigation to proof the light‐induced formation of CF_3_ radicals by trapping the radicals with c) TEMPO **4 a** or d) α‐methylstyrene **5 a**.

In order to gain further insight into this polymer‐supported photocatalytic process, we performed several control experiments (Table 1). First, we conducted the trifluoromethylation of **2 a** in the presence of photoredox‐inactive **P4**, which has no PTH catalyst incorporated (Table 1, entry 1). Neither product formation nor conversion of Togni's reagent **1 a** were observed. The same outcome was obtained without the use of any SCPN or running the reaction in the dark (Table 1, entries 2 and 3). Thus, successful product formation only transpires by exciting photoredox‐active SCPNs (**P1**–**P3**) with an appropriate light source. As such sunlight also proved to be a suitable energy source to fuel the reaction instead of UV light, thereby contributing to energy‐efficient, sustainable chemistry (Table 1, entry 4, Figure 5a). In addition, our polymeric system displays catalytic activity even in the presence of air oxygen as the high reduction power of PTH photocatalysts primarily stems from the excited singlet state and not from the oxygen‐sensitive triplet state (Table 1, entry 5).[^36^, ^58^] This tolerance to molecular oxygen streamlines the operational effort and represents a premise for potential biological applications.

**Table 1:** Control experiments to investigate the applicability of this aqueous trifluoromethylation method

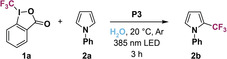		
Entry	Deviation from optimized conditions^[a]^	Formation of **2 b**
0	–	93 %
1	**P4**	0 %
2	no SCPN	0 %
3	no light	0 %
4	sunlight (7 h)	63 %
5	not degassed (18 h)	65 %
6	in THF‐*d*_6_	50 %
7	in CDCl_3_	65 %
8	in ACN‐*d*_3_	68 %
9	**1 d** in ACN‐*d*_3_	50 %
10	**1 d**/**P4**	66 %

[a] Formation of side product **2 c** was low (≤4 %) in all cases.

To support the significance of creating amphiphilic nanoparticles and providing hydrophobic compartments in an aqueous environment, we ran the reaction in various organic solvents, in which the hydrophobic collapse of the polymer chains to nanoreactors is restricted. Indeed, lower conversions to the product **2 b** between 50–68 % were determined by changing the medium to deuterated tetrahydrofuran, chloroform, or acetonitrile while keeping the other reaction parameters constant (Table 1, entries 6–8). Another experiment was executed with small molecule catalyst **1 d** to compare with the performance of polymer‐bound PTH (**P3**) at equal catalyst loadings in acetonitrile (Table 1, entry 9). Since a slightly diminished **2 b** formation of 50 % was observed, it can be concluded that the catalytic efficiency of PTH is not compromised by the polymer support.

Following up on this, we wanted to examine if the covalent attachment of PTH to the polymer backbone (**P3**) is beneficial compared to the loose encapsulation of catalyst **1 d** within the hydrophobic interior of catalyst‐free SCPNs (**P4**). Thus, we dissolved **1 d** and 10 mg of **P4** in dichloromethane, removed the solvent under vacuum, and dissolved the mixture again in water after adding the reagents **1 a** and **2 a**. After three hours of irradiation, a conversion of 66 % to product **2 b** was determined (Table 1, entry 10), which is significantly lower than when **P3** is applied. This difference can be explained by the competition of catalyst **1 d** with the other reagents for the hydrophobic space within the nanoparticles. Consequently, anchoring the photocatalyst to the polymeric backbone not only results in a more reliable and higher catalytic activity but also enables facile reusing of the nanoparticles for consecutive runs. As catalyst leaking is prevented, the organic compounds can conveniently be extracted from the aqueous mixture with diethyl ether while the catalyst‐loaded SCPNs remain in the water phase. A recycling study was performed to demonstrate that the same aqueous solution containing polymer **P3** can be used for the serial preparation of compound **3 q** (Figure 5b). The extent of product formation stayed roughly constant over the course of three iterations, thus highlighting the high robustness and recyclability of our catalytic system.

Lastly, we investigated if the direct C−H trifluoromethylation proceeds via a light‐induced generation of CF_3_ radicals (a possible reaction mechanism is depicted in Figure S15). Hence, we added the bench‐stable radical TEMPO **4 a** to the aqueous reaction mixture containing reagents **1 a** and **2 a** (Figure 5c). As expected, the produced CF_3_ radicals preferably recombined with TEMPO to form the corresponding adduct **4 b** while product **2 b** was not detected. Interested to investigate if the CF_3_ radicals also add to alkene groups in aqueous conditions, we employed three equivalents of α‐methylstyrene **5 a** as the substrate (Figure 5d). After 7 h of UV light illumination we observed the hydroxytrifluoromethylation product **5 b**.^[64]^ In addition, the minor product **5 c** was detected, which formed by elimination of water from **5 b**. In this case water not only served as the solvent but also actively participated in the reaction as a nucleophile.

## Conclusions

The *N‐*phenylphenothiazine‐decorated amphiphilic polymers developed here provide versatile and recyclable nanoreactors in water that function efficiently and selectively in the light‐induced direct C−H trifluoromethylation of a wide range of substrates including biologically relevant compounds. Optimization of the reaction conditions revealed that the highest efficacy and selectivity was achieved at low catalyst loadings (e. g., **P3** with only 5 % PTH attached gave the best results). Interestingly, the efficiency of the trifluoromethylation reaction was improved by lowering the irradiance of the used LED to one quarter of the power initially used (which corresponds to only 5 % of the maximum LED output power). The application of low intensity light at ambient temperature strongly increases the energy efficiency of our protocol. Light intensity is a crucial parameter in optimizing photocatalytic processes, but is still largely underrepresented in literature.[^67^, ^68^] As the reaction partners and covalently bound photocatalysts gather in a confined space within the nanoparticles under high local concentrations, the production of CF_3_ radicals and thus the formation of the desired products is significantly stimulated. Consequently, our aqueous photocatalytic method requires significantly shorter reaction times (t=3 h) compared to literature reports on direct C−H trifluoromethylations that operate in homogenous solutions in organic solvents (t≤2 d),[^69^, ^70^, ^71^] is faster than trifluoromethylations in water using non‐photocatalytic methods,^[55]^ or affords better conversions when using the same trifluoromethylating reagent in DMSO.^[72]^ In addition, compared to the procedures that introduce CF_3_ groups via a photocatalytic trifluoromethylation in water,[^25^, ^56^, ^57^] our approach is faster (3 h vs. >24 h), amenable to the use of sunlight, does not require precious and rare transition metals, and is fully recyclable.

Mechanistic studies suggest the in situ formation of highly reactive CF_3_ radicals upon light illumination. In the presence of a styrene derivative, water also participates as a nucleophile to form the hydroxytrifluoromethylated product **5 b**. Overall, this study contributes to environmentally friendly photon‐driven catalytic processes since potentially harmful organic solvents as well as metal catalysts are avoided. Note that upscaling the reaction, for instance with the aid of flow reactors,^[15]^ would facilitate an improved workup procedure (e. g., filtration of solid products instead of extraction). In addition, the reusability of the catalytic system and the possibility of employing sunlight renders this trifluoromethylation method sustainable, which entails a vast potential for late‐stage modifications of pharmaceutical or agrochemical compounds.

## Experimental Section

A 1.5 mL glass vial with septum screw cap was charged with trifluoromethylation reagent (30 μmol, 1.0 equiv), substrate (45 μmol, 1.5 equiv), and SCPN (10 mg) and 0.8 mL of deionized water was added. After sealing the capped vial with parafilm and degassing the aqueous phase with argon for 5 min, the reaction mixture was illuminated with a focused 385 nm LED for the indicated time while being stirred and cooled with compressed air. Afterwards, trifluorotoluene (30 μmol, 1.0 equiv) as the internal ^19^F NMR standard was added and the mixture was extracted three times with 0.5 mL of deuterated chloroform. Product formation was determined via quantitative ^19^F NMR spectroscopy and verified by GC‐MS if applicable.

## Author Contributions

F.E. synthesized, isolated, and characterized the compounds. F.E. performed the catalysis experiments. F.E and A.R.A.P. designed the study, wrote and edited the manuscript.

## Conflict of interest

The authors declare no conflict of interest.

1

## Supporting information

As a service to our authors and readers, this journal provides supporting information supplied by the authors. Such materials are peer reviewed and may be re‐organized for online delivery, but are not copy‐edited or typeset. Technical support issues arising from supporting information (other than missing files) should be addressed to the authors.

Supporting Information

## Data Availability

The data that support the findings of this study are available in the supplementary material of this article.
