# Supplementary material for: Direct C−H Trifluoromethylation of (Hetero)Arenes in Water Enabled by Organic Photoredox‐Active Amphiphilic Nanoparticles
Source: Chemistry. 2022 Jul 28;28(52):e202201322. doi: 10.1002/chem.202201322 (PMC9544737; doi:10.1002/chem.202201322)
Supplement: Supplementary file 1 — Supporting Information [file CHEM-28-0-s001.pdf]

Chemistry–A European Journal

Supporting Information

Direct C–H Trifluoromethylation of (Hetero)Arenes in Water Enabled by Organic Photoredox-Active Amphiphilic Nanoparticles

Fabian Eisenreich* and Anja R. A. Palmans*

Contents

1. General methods and equipment 1

2. Synthesis 2

3. Polymer characterization 6

4. Photoredox catalysis experiments 14

5. NMR spectra of final compounds 38

6. References 42

1. General methods and equipment

Solvents and commercial starting materials were used as received. Jeffamine® M-1000 polyetheramine was provided by Huntsman. All solvents were purchased from Biosolve and the deuterated solvents were purchased from Cambridge Isotopes Laboratories. In case of using dry solvents, a MBraun solvent purification system (MB SPS-800) was employed. Reactions were monitored by thin layer chromatography (TLC) carried out on silica gel plates (Merck 60F-254) using UV light for visualization. Automated column chromatography was carried out with Biotage Isolera® One using Biotage Silica cartridges and eluents as specified. NMR spectra were recorded on a Bruker 400 MHz Ultrashield spectrometer (400 MHz for ^{1}H, 100 MHz for ^{13}C, and 376 MHz for ^{19}F) at 25 °C using residual protonated solvent signals as internal standards for ^{1}H and ^{13}C spectra (^{1}H: δ($CDCl_3$) = 7.26 ppm, δ(DMSO-d_6) = 2.50 ppm, δ(D_2O) = 4.79 ppm; ^{13}C: δ($CDCl_3$) = 77.16 ppm, δ(DMSO-d_6) = 39.52 ppm). The splitting patterns are abbreviated as follows: singlet (s), doublet (d), triplet (t), quartet (q), multiplet (m), and broad (br). Matrix assisted laser desorption/ ionization time of flight mass spectra (MALDI-TOF-MS) was acquired using a Bruker Autoflex Speed MALDI-TOF using α-cyano-4-hydroxycinnamic acid (CHCA) or *trans*-2-[3-(4-*tert*-butylphenyl)-2-methyl-2-propenylidene]malononitrile (DCTB) as matrices. For poly(penta-fluorophenyl acrylate), size exclusion chromatography (SEC) measurements were performed on a Shimadzu Prominence-i LC-2030C 3D with a Shimadzu RID-20A refractive index detector. As elution, THF (flow = 1 mL min^{-1}) was used operating at 40 °C with a mixed-C and mixed-D column combined in series (exclusion limit = 2.000.000 g mol^{-1}; 7.5 mm i.d. × 300 mm) calibrated with poly(styrene) (Polymer Laboratories). For the post-functionalized amphiphilic polymers, SEC measurements were performed on a PL-GPC-50 plus from Polymer Laboratories (Varian Inc. Company) with a refractive index detector. As elution, dimethylformamide containing 10 mM LiBr (flow = 1 mL min^{-1}) was used operating at 50 °C with a Shodex GPC-KD-804 column (exclusion limit = 400.000 Da; 0.8 cm i.d. × 300 mm) calibrated with poly(ethyleneoxide) (Polymer Laboratories). SEC measurements in PBS (pH = 7.4) were carried out on a Shimadzu CBM-20A System at 20 °C equipped with a Shimadzu RID-10A RI detector, SIL-20A autosampler, and 2 LC-20AD pumps on a Shodex OHpak SB-804 HQ column (exclusion limit = 1000 kDa, i.d. = 0.8 cm, L = 300 mm) with a TSKgel SWXL type guard column (i.d. = 0.6 cm, L = 40 mm), with PBS as an eluent at a constant flow rate of 0.8 mL min^{-1}. The column was calibrated against poly(ethylene oxide) (Polymer Laboratories). For dialysis, a Standard RC Tubing was used from Spectra/Por® with a molecular weight cut-off (MWCO) of 6–8 kDa. Dynamic light scattering (DLS) measurements were performed using a Malvern µV Zetasizer equipped with an 830 nm laser and a scattering angle of 90°. Samples were prepared at 1 mg/mL and held in Sarstedt UV-transparent disposable cuvettes with a path length of 10 x 2 mm. Measurements were analyzed using Zetasizer software provided by Malvern Instruments to derive the correlation functions and the distributions of the hydrodynamic diameter. UV/vis spectroscopy experiments were performed on a JASCO V-650 spectrometer at 20 °C using a JASCO CTU-100 Circulating Thermoset Unit. For photoredox catalysis experiments, light-emitting diodes (LEDs) from Thorlabs were used (385LP1) equipped with a SM2F32-A collimator to focus the light. Irradiance *E* was measured with Radiometer RM12 from Opsytec Dr. Gröbel.

2. Synthesis

10-Phenylphenothiazine (1d)

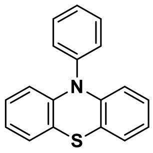

Under inert conditions, iodobenzene (0.27 mL, 2.4 mmol, 1.2 eq.), potassium *tert*-butoxide (292 mg, 2.6 mmol, 1.3 eq.), $Pd_2(dba)_3$ (55 mg, 0.06 mmol, 0.03 eq.), and tri-*tert*-butylphosphonium tetrafluoroborate (35 mg, 0.12 mmol, 0.06 eq.) were added to a solution of phenothiazine (399 mg, 2.0 mmol, 1.0 eq.) in 4 mL of dry toluene. The reaction mixture was stirred at 110 °C for 18 h and subsequently cooled to room temperature. The mixture was diluted with dichloromethane and filtered through a pad of celite. The crude product was purified by column chromatography (SiO_2, heptane/ethyl acetate) to yield the desired product **1d** (440 mg, 1.6 mmol, 80% yield) as a white solid.

^{1}H-NMR (400 MHz, $CDCl_3$, 25 °C): δ = 7.61 (t, *J* = 7.6 Hz, 2H, Ar-CH), 7.48 (t, *J* = 7.5 Hz, 2H, Ar-CH), 7.40 (d, *J* = 7.6 Hz, 2H, Ar-CH), 7.03 (dd, *J* = 7.2 Hz, 2.0 Hz, 2H, Ar-CH), 6.84 (m, 4H, Ar-CH), 6.21 (d, *J* = 7.7 Hz, 2H, Ar-CH) ppm.
^{13}C-NMR (100 MHz, $CDCl_3$, 25 °C): δ = 144.4, 141.1, 131.0, 130.9, 128.3, 127.0, 126.8, 122.6, 120.3, 116.2 ppm.
MALDI-ToF-MS (m/z) calc. for $C_{18}H_{13}NS^{\cdot+}$ $[M]^{\cdot+}$: 275.08, found: 275.11

tert-Butyl (4-bromophenethyl)carbamate (A1)

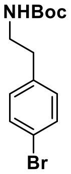

A solution of 4-bromophenethylamine (3.0 g, 15.0 mmol, 1.0 eq.) in 200 mL of dry tetrahydrofuran was cooled in an ice bath and di-*tert*-butyl dicarbonate (5.6 g, 25.5 mmol, 1.7 eq.) was added portionwise. The mixture was allowed to warm up to room temperature and stirred for 18 h. After removal of the solvent under reduced pressure, the crude product was purified by flash column chromatography (SiO_2, heptane/ethyl acetate) to yield the desired product **A1** (4.0 g, 13.3 mmol, 89% yield) as a white solid compound. NMR signals are in accordance to literature.[1]

^{1}H-NMR (400 MHz, $CDCl_3$, 25 °C): δ = 7.42 (m, 2H, Ar-CH), 7.06 (d, *J* = 8.2 Hz, 2H, Ar-CH), 4.53 (br, 1H, NH), 3.34 (q, *J* = 6.8 Hz, 2H, CH_2), 2.75 (t, *J* = 6.8 Hz, 2H, CH_2), 1.43 (s, 9H, CH_3) ppm.
^{13}C-NMR (100 MHz, $CDCl_3$, 25 °C): δ = 155.9, 138.1, 131.7, 130.7, 120.4, 79.5, 41.7, 35.8, 28.5 ppm.
MALDI-ToF-MS (m/z) calc. for $C_{13}H_{18}BrNNaO_2^+$ $[M+Na]^+$: 322.04, found 322.17.

***tert*-Butyl (4-(10H-phenothiazin-10-yl)phenethyl)carbamate (A2)**

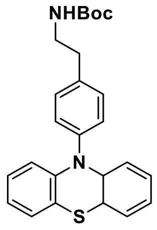

Under inert conditions, phenothiazine (1.2 mg, 6.0 mmol, 1.0 eq.), compound **A1** (1.8 mg, 6.0 mmol, 1.0 eq.), sodium *tert*-butoxide (750 mg, 7.8 mmol, 1.3 eq.), RuPhos precat (98 mg, 120 µmol, 0.02 eq.), and RuPhos (56 mg, 120 µmol, 0.02 eq.) were suspended in 6 mL of dry dioxane and stirred at 100 °C for 6 h. After cooling to room temperature, the mixture was diluted with chloroform, washed with brine, and dried over $MgSO_4$. The solvent was removed and the crude product was purified by flash column chromatography (SiO_2, heptane/ethyl acetate) followed by recrystallization from a heptane/ethyl acetate mixture to yield the desired product **A2** (1.2 mg, 2.9 mmol, 48% yield) as an off-white solid compound.

^{1}H-NMR (400 MHz, $CDCl_3$, 25 °C): δ = 7.42 (d, *J* = 8.2 Hz, 2H, Ar-CH), 7.32 (d, *J* = 8.2 Hz, 2H, Ar-CH), 7.01 (dd, *J* = 7.3, 1.9 Hz, 2H, Ar-CH), 6.91 – 6.74 (m, 4H, Ar-CH), 6.20 (dd, *J* = 8.0, 1.2 Hz, 2H, Ar-CH), 4.65 (br, 1H, NH), 3.47 (q, *J* = 6.7 Hz, 2H, CH_2), 2.90 (t, *J* = 7.0 Hz, 2H, CH_2), 1.46 (s, 9H, CH_3) ppm.
^{13}C-NMR (100 MHz, $CDCl_3$, 25 °C): δ = 156.0, 144.4, 139.4, 131.2, 131.1, 126.9, 126.8, 122.5, 120.3, 116.1, 79.5, 41.8, 36.3, 28.6 ppm. (one aromatic carbon signal is missing due to low intensity)
MALDI-ToF-MS (m/z) calc. for $C_{25}H_{26}N_2O_2S^{\cdot+}$ $[M]^{\cdot+}$: 418.17, found: 418.18

2-(4-(10H-Phenothiazin-10-yl)phenyl)ethan-1-amine (A3)

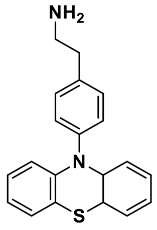

Compound **A2** (330 mg, 0.79 mmol, 1.0 eq.) was dissolved in 3 mL of 4 M HCl solution in dioxane. After stirring the mixture at room temperature for 2 h, full conversion to the deprotected product was observed by TLC. The mixture was treated with 30 mL of NaOH solution (3 M) and stirred for 2 h. Subsequently, the aqueous mixture was extracted with dichloromethane (3x 10 mL) and the combined organic phases were dried over $MgSO_4$. Removal of the solvent yielded the desired product **A3** (250 mg, 0.79 mmol, 99% yield) as an off-white solid.

^{1}H-NMR (400 MHz, DMSO-d_6, 25 °C): δ = 7.48 (d, *J* = 8.1 Hz, 2H, Ar-CH), 7.31 (d, *J* = 8.1 Hz, 2H, Ar-CH), 7.05 (dd, *J* = 7.5, 1.3 Hz, 2H, Ar-CH), 6.91 (td, *J* = 7.8, 1.3 Hz, 2H, Ar-CH), 6.83 (td, *J* = 7.8, 1.3 Hz, 2H, Ar-CH), 6.15 (dd, *J* = 8.2, 1.3 Hz, 2H, Ar-CH), 2.87 (t, *J* = 7.0 Hz, 2H, CH_2), 2.76 (t, *J* = 7.0 Hz, 2H, CH_2), 1.70 (br, 2H, NH_2) ppm.
^{13}C-NMR (100 MHz, DMSO-d_6, 25 °C): δ = 143.8, 140.9, 137.9, 131.2, 130.2, 127.2, 126.6, 122.5, 119.0, 115.8, 43.4 ppm. (one aliphatic carbon signal is missing)
MALDI-ToF-MS (m/z) calc. for $C_{20}H_{18}N_2S^{\cdot+}$ $[M]^{\cdot+}$: 318.12, found: 318.13

Poly(pentafluorophenyl acrylate) (A4)

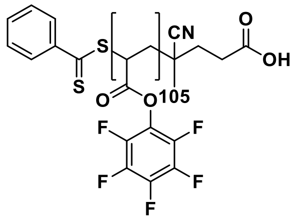

Under inert conditions, pentafluorophenyl acrylate (6.2 g, 26.5 mmol, 148 eq.), cyano-4-(phenyl-carbonothioylthio)pentanoic acid (49.3 mg, 0.177 mmol, 1.0 eq.), and azobisisobutyronitrile (4.4 mg, 26.5 µmol, 0.15 eq.) were dissolved in 6 mL of dry dioxane and placed in a pre-heated oil bath at 80 °C. After 3 h, a sample was taken from the mixture and the monomer consumption was determined by ^{19}F NMR spectroscopy to be 71%, which corresponds to a degree of polymerization of 105. Subsequently, the polymerization mixture was quenched by exposing it to oxygen and the polymer was precipitated into 1 L of ice-cold pentane. The polymer was filtered off and dried in a vacuum oven at 50 °C for 18 h. As a result, 3.8 g of the desired polymer **A4** was obtained as a light reddish solid compound. SEC (THF): M_n = 18 kg/mol, $Đ$ = 1.19.

End-cap modification of poly(pentafluorophenyl acrylate) (A5)

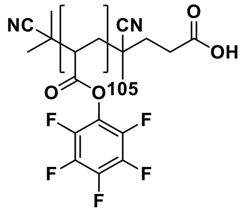

Under inert conditions, poly(pentafluorophenyl acrylate) **A4** (3.0 g, 158 µmol, 1.0 eq.), azobisisobutyronitrile (522 mg, 3.18 mmol, 20 eq.), and lauroyl peroxide (127 mg, 319 µmol, 2.0 eq.) were dissolved in 5 mL of dry dioxane and heated to 80 °C for 2 hours. Afterwards, the solvent was removed and the residue re-dissolved in 5 mL of chloroform, followed by precipitating the polymer three times into 0.5 L of ice-cold pentane and drying it in a vacuum oven at 50 °C for 18 h. As a result, 2.3 g of the desired polymer **A5** was obtained as a white solid compound and directly used for side-group modification without further characterization.

Polymer P1

Under inert conditions, poly(pentafluorophenyl acrylate) **A5** (100 mg, 4.2 µmol, 1.0 eq.) was dissolved in 3 mL of dry tetrahydrofuran. At first, compound **A3** (26.5 mg, 83.2 µmol, 20.0 eq.) was added, followed by Jeffamine® (0.5 g, 0.42 mmol, 100.0 eq.). After each addition step, the mixture was stirred at 50 °C for 18 h and the conversion was subsequently measured by ^{19}F NMR spectroscopy before the next reagent was added (see Figure S2). The final fully functionalized polymer was purified by dialysis, first in tetrahydrofuran and then in methanol. After removal of the solvent and drying in a vacuum oven at 50 °C for 18 h, 350 mg of **P1** was obtained as a waxy solid compound. SEC(DMF): M_n = 31.2 kg/mol, $Đ$ = 1.25. SEC(PBS): M_n = 25.2 kg/mol, $Đ$ = 1.30. See Figure S8 for ^1H NMR spectrum in $CDCl_3$.

Polymer P2

Under inert conditions, poly(pentafluorophenyl) acrylate **A5** (100 mg, 4.2 µmol, 1.0 eq.) was dissolved in 3 mL of dry tetrahydrofuran. At first, compound **A3** (13.3 mg, 41.6 µmol, 10.0 eq.) was added, followed by dodecylamine (7.7 mg, 41.6 µmol, 10.0 eq.) and Jeffamine® (0.5 g, 0.42 mmol, 100.0 eq.). After each addition step, the mixture was stirred at 50 °C for 18 h and the conversion was subsequently measured by ^{19}F NMR spectroscopy before the next reagent was added (see Figure S3). The final fully functionalized polymer was purified by dialysis, first in tetrahydrofuran and then in methanol. After removal of the solvent and drying in a vacuum oven at 50 °C for 18 h, 350 mg of **P2** was obtained as a waxy solid compound. SEC(DMF): M_n = 29.9 kg/mol, *Đ* = 1.17. SEC(PBS): M_n = 19.7 kg/mol, *Đ* = 1.27. See Figure S9 for ^{1}H NMR spectrum in $CDCl_3$.

Polymer P3

Under inert conditions, poly(pentafluorophenyl) acrylate **A5** (420 mg, 17.5 µmol, 1.0 eq.) was dissolved in 12 mL of dry tetrahydrofuran. At first, compound **A3** (28.0 mg, 87.4 µmol, 5.0 eq.) was added, followed by dodecylamine (48.7 mg, 262 µmol, 15.0 eq.) and Jeffamine® (2.1 g, 1.75 mmol, 100.0 eq.). After each addition step, the mixture was stirred at 50 °C for 18 h and the conversion was subsequently measured by ^{19}F NMR spectroscopy before the next reagent was added (see Figure S4). The final fully functionalized polymer was purified by dialysis, first in tetrahydrofuran and then in methanol. After removal of the solvent and drying in a vacuum oven at 50 °C for 18 h, 1.5 g of **P3** was obtained as a waxy solid compound. SEC(DMF): M_n = 28.6 kg/mol, *Đ* = 1.19. SEC(PBS): M_n = 12.1 kg/mol, *Đ* = 1.29. See Figure S10 for ^{1}H NMR spectrum in $CDCl_3$.

Polymer P4

Under inert conditions, poly(pentafluorophenyl) acrylate **A5** (500 mg, 20.8 µmol, 1.0 eq.) was dissolved in 10 mL of dry tetrahydrofuran. At first, dodecylamine (77.3 mg, 417 µmol, 20.0 eq.) was added, followed by Jeffamine® (2.0 g, 2.08 mmol, 100.0 eq.). After each addition step, the mixture was stirred at 50 °C for 18 h and the conversion was subsequently measured by ^{19}F NMR spectroscopy before the next reagent was added (see Figure S5). The final fully functionalized polymer was purified by dialysis, first in tetrahydrofuran and then in methanol. After removal of the solvent and drying in a vacuum oven at 50 °C for 18 h, 1.8 g of **P4** was obtained as a waxy solid compound. SEC(DMF): M_n = 33.7 kg/mol, *Đ* = 1.28. SEC(PBS): M_n = 16.2 kg/mol, *Đ* = 1.32. See Figure S11 for ^{1}H NMR spectrum in $CDCl_3$.

3. Polymer characterization

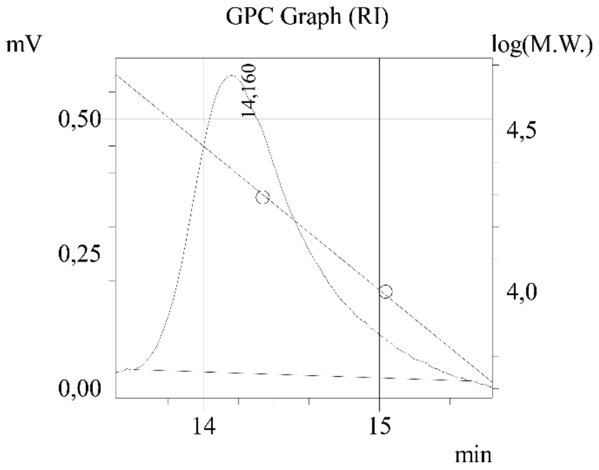

Figure S1: Size exclusion chromatogram of polymer **A5** in THF (c = 1 mg/mL).

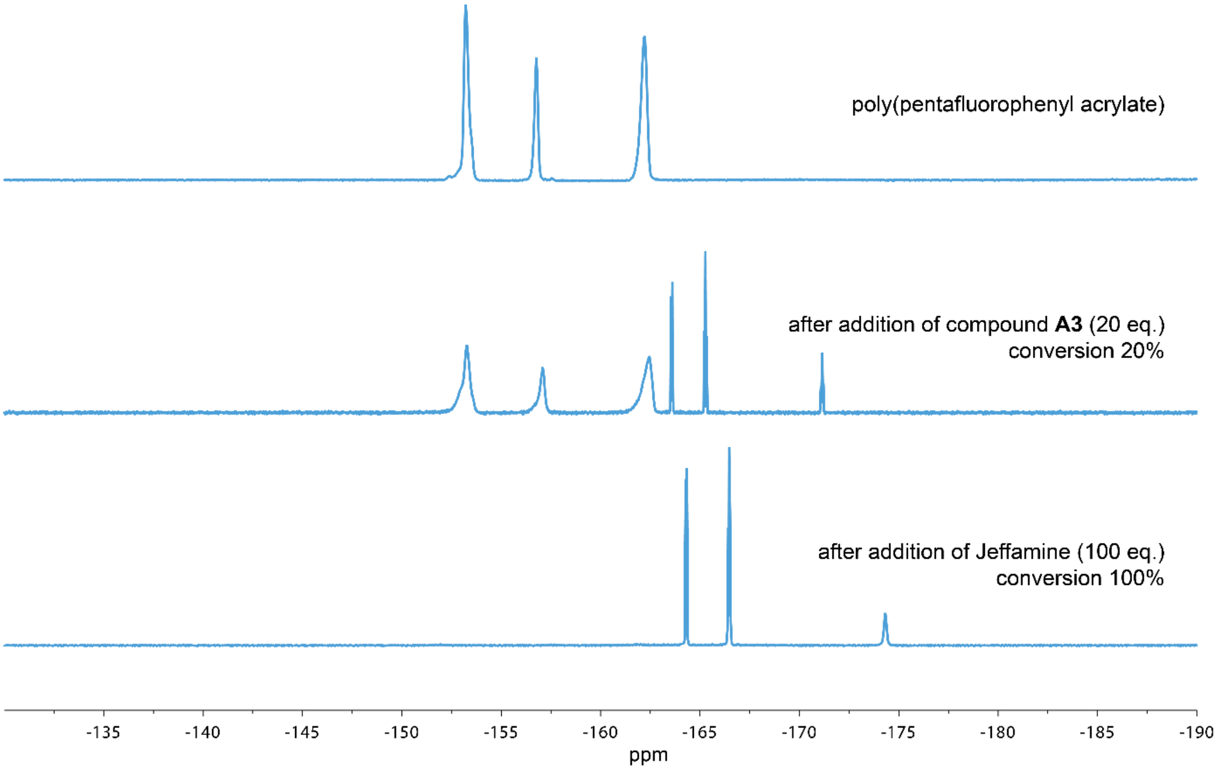

Figure S2: ^{19}F NMR spectra (376 MHz, $CDCl_3$, 25 °C) recorded before post-functionalization (top) and after each addition step during the synthesis of polymer **P1**. The degree of functionalization was determined based on the ratio of pentafluorophenol integrated into the polymer backbone (broad signals) and free pentafluorophenol (sharp signals).

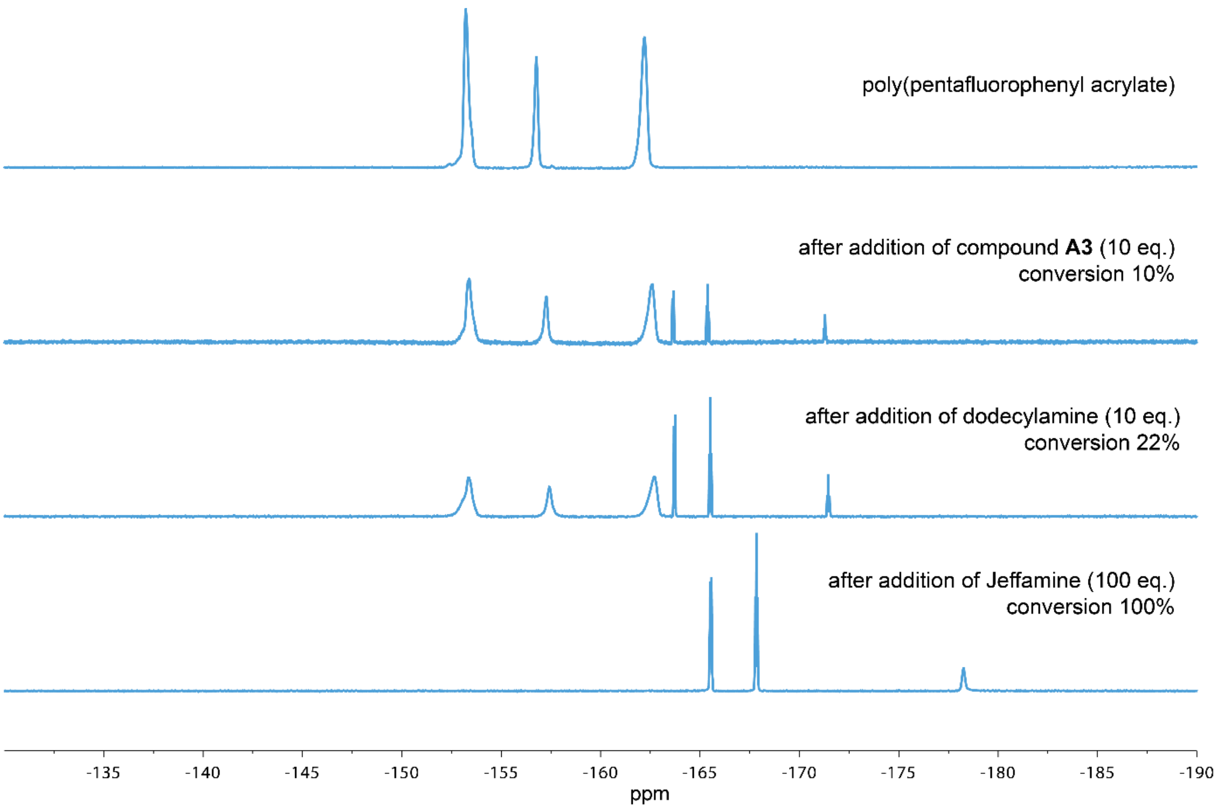

Figure S3: ^{19}F NMR spectra (376 MHz, $CDCl_3$, 25 °C) recorded before post-functionalization (top) and after each addition step during the synthesis of polymer **P2**. The degree of functionalization was determined based on the ratio of pentafluorophenol integrated into the polymer backbone (broad signals) and free pentafluorophenol (sharp signals).

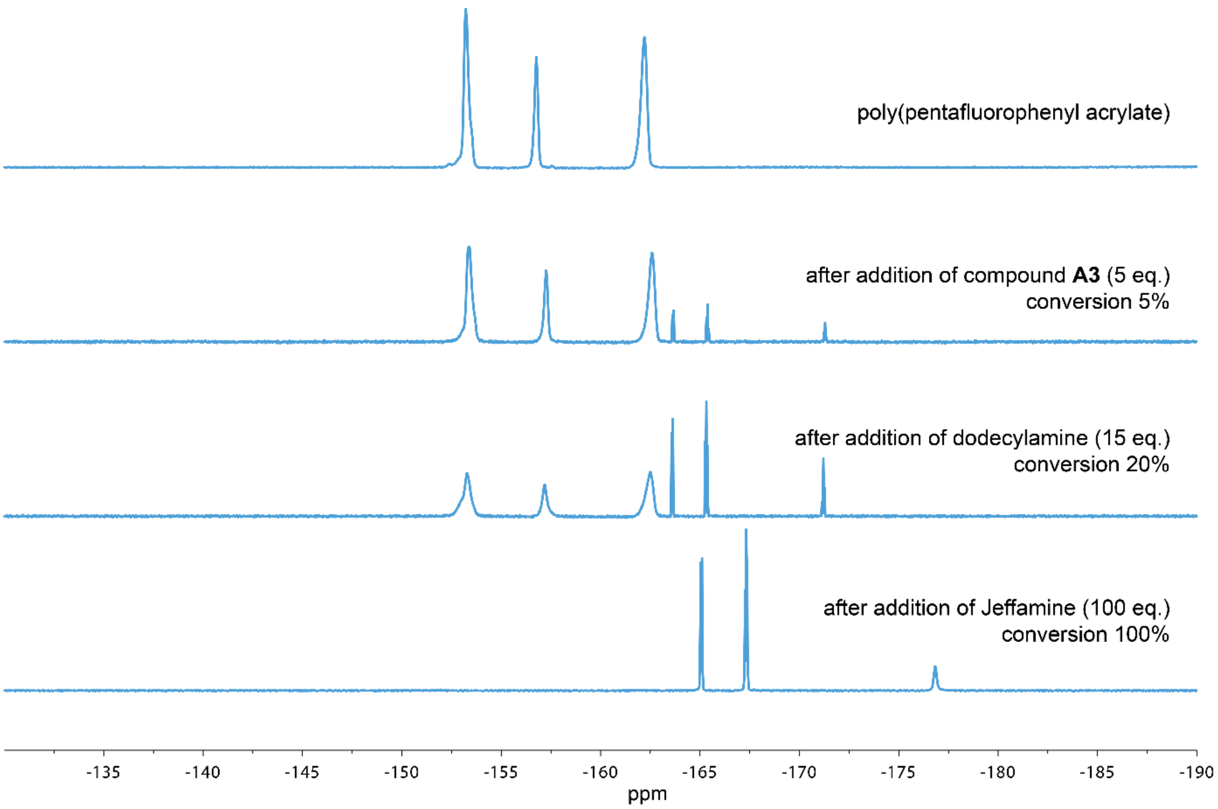

Figure S4: ^{19}F NMR spectra (376 MHz, $CDCl_3$, 25 °C) recorded before post-functionalization (top) and after each addition step during the synthesis of polymer **P3**. The degree of functionalization was determined based on the ratio of pentafluorophenol integrated into the polymer backbone (broad signals) and free pentafluorophenol (sharp signals).

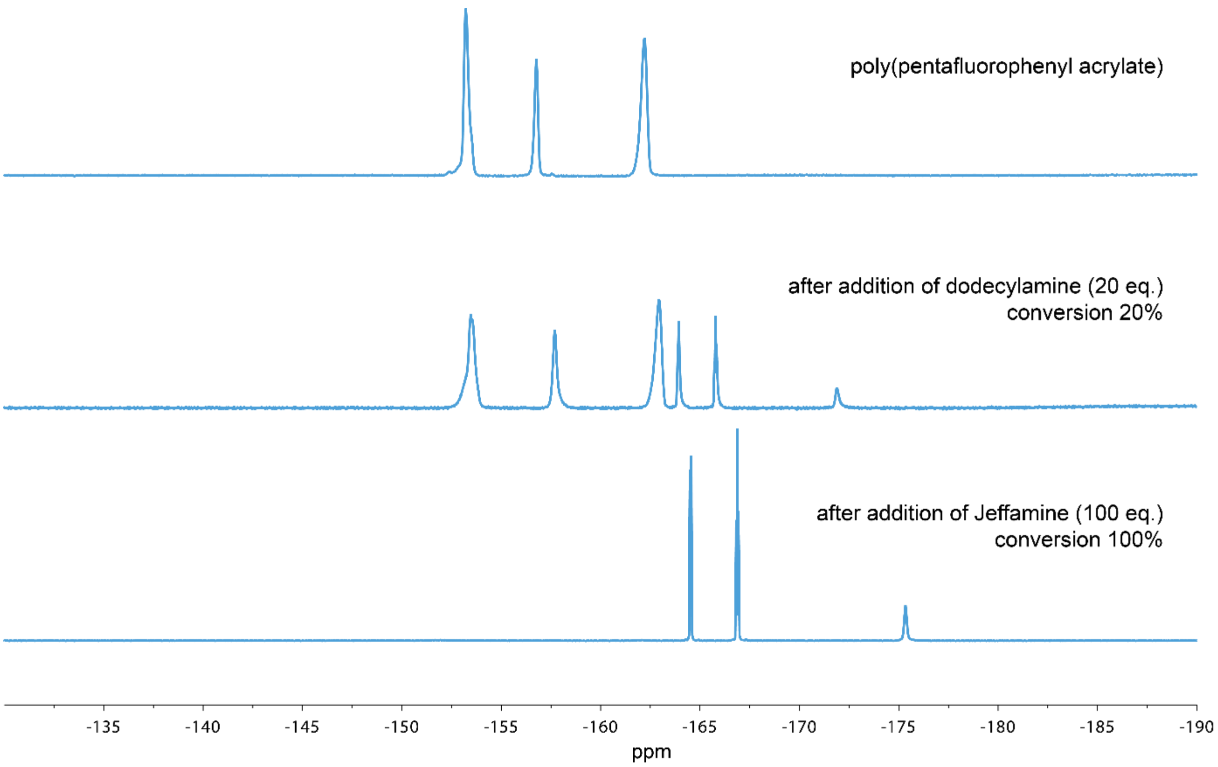

Figure S5: ^{19}F NMR spectra (376 MHz, $CDCl_3$, 25 °C) recorded before post-functionalization (top) and after each addition step during the synthesis of polymer **P4**. The degree of functionalization was determined based on the ratio of pentafluorophenol integrated into the polymer backbone (broad signals) and free pentafluorophenol (sharp signals).

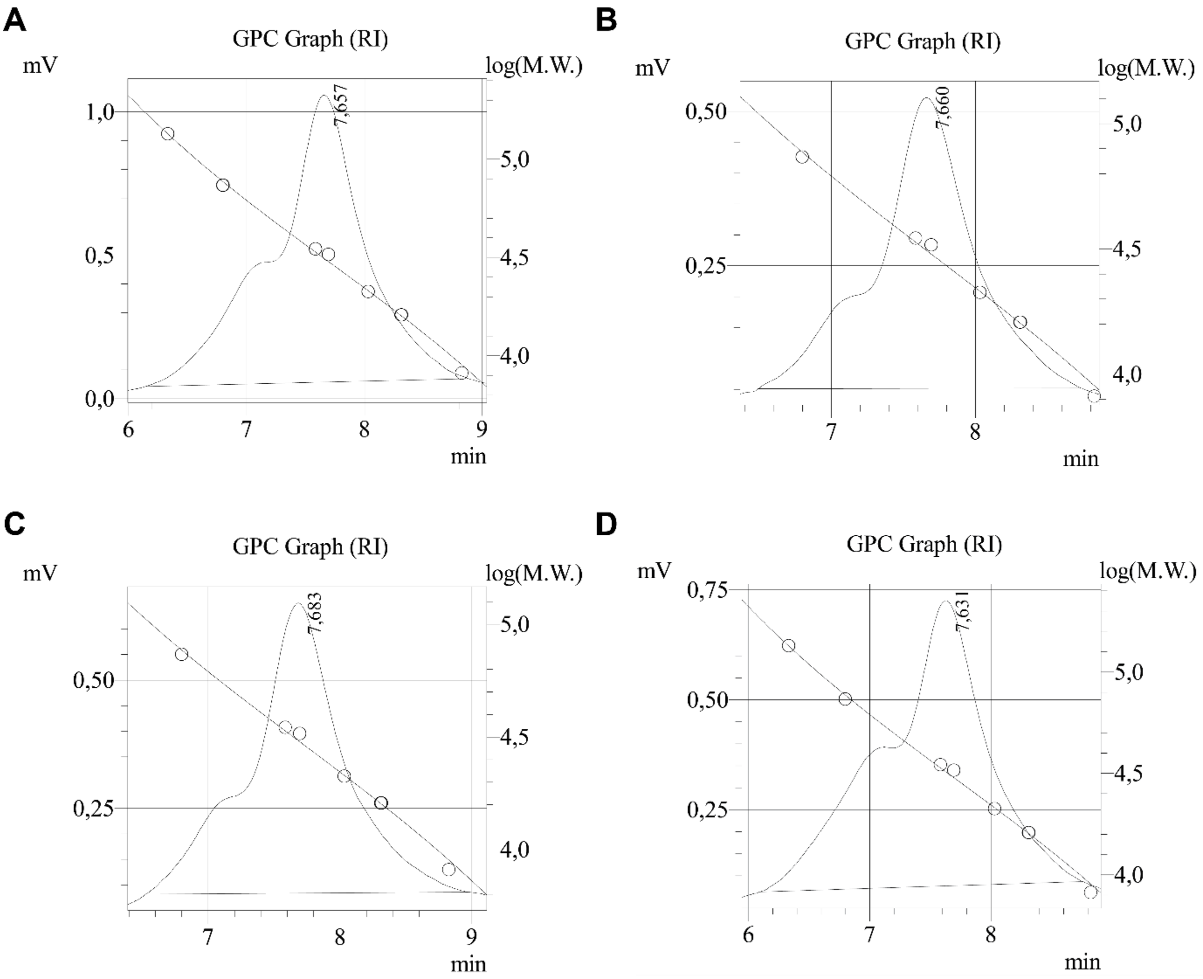

Figure S6: Size exclusion chromatograms of polymers **P1**–**P4** (**A**–**D**) in DMF with LiBr (*c* = 1 mg/mL). The shoulders at a retention time of around 7 min originate from higher molecular weight polymers that form as a radical recombination product during the end-cap modification step (synthesis of **A5**).

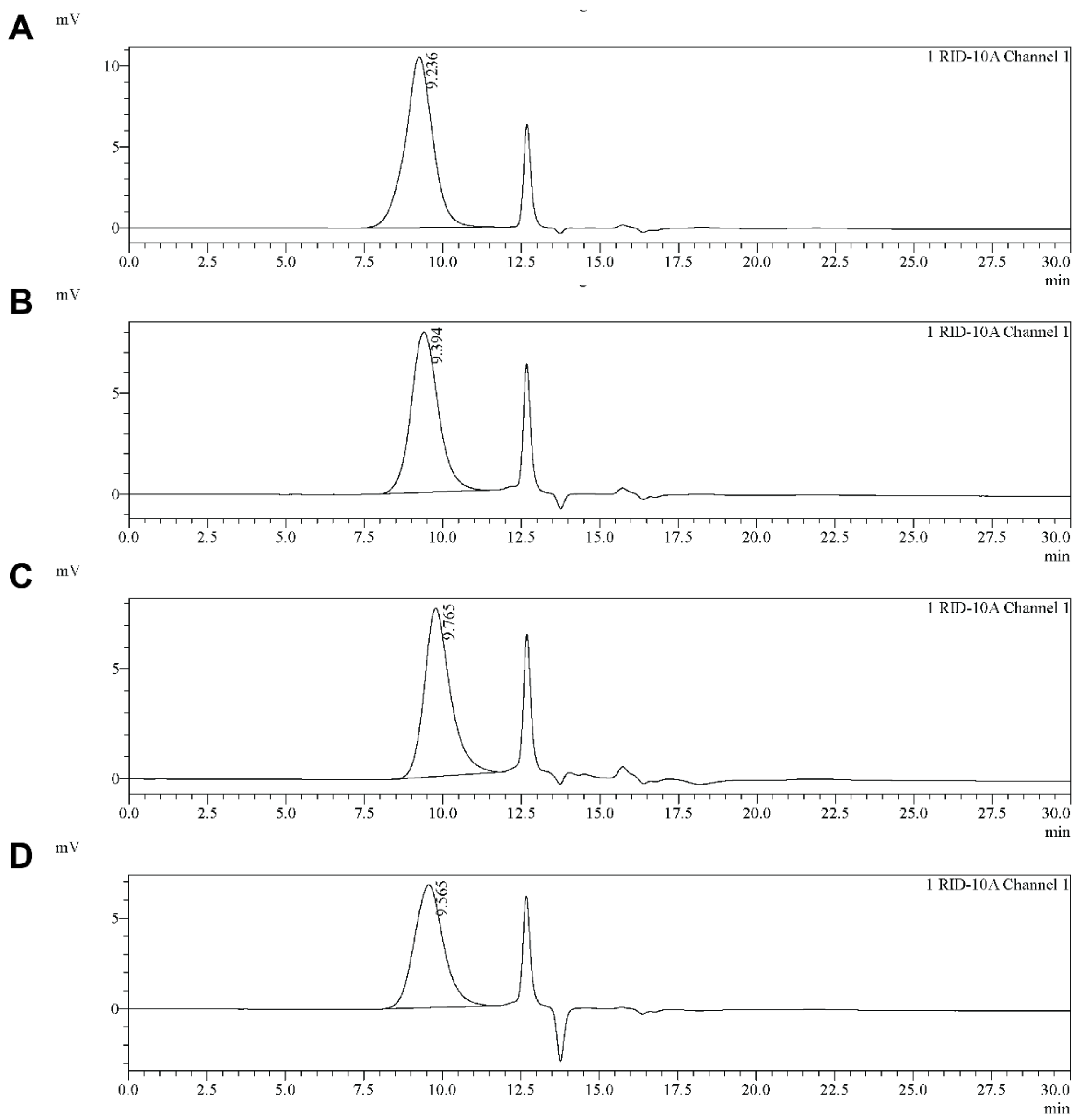

Figure S7: Size exclusion chromatograms of polymers **P1–P4** (**A–D**) in PBS (pH = 7.4, *c* = 1 mg/mL).

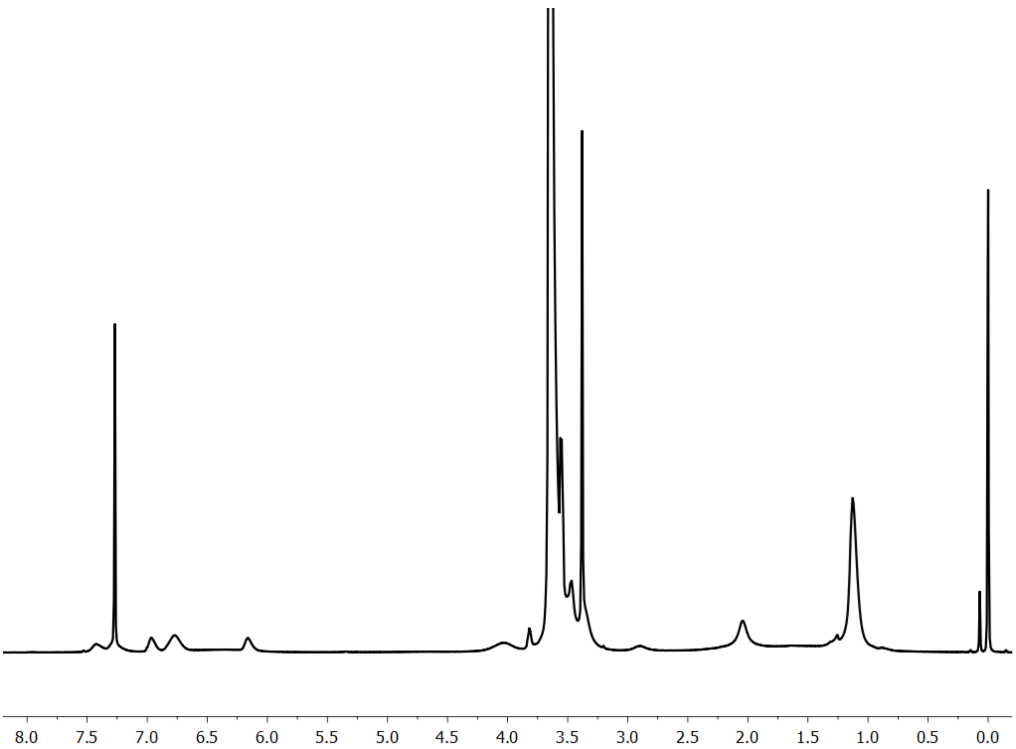

Figure S8: ^{1}H NMR spectrum of polymer **P1** (400 MHz, $CDCl_3$, 25 °C). Signals for the incorporated PTH catalyst are visible in the aromatic region between 6.0–7.5 ppm.

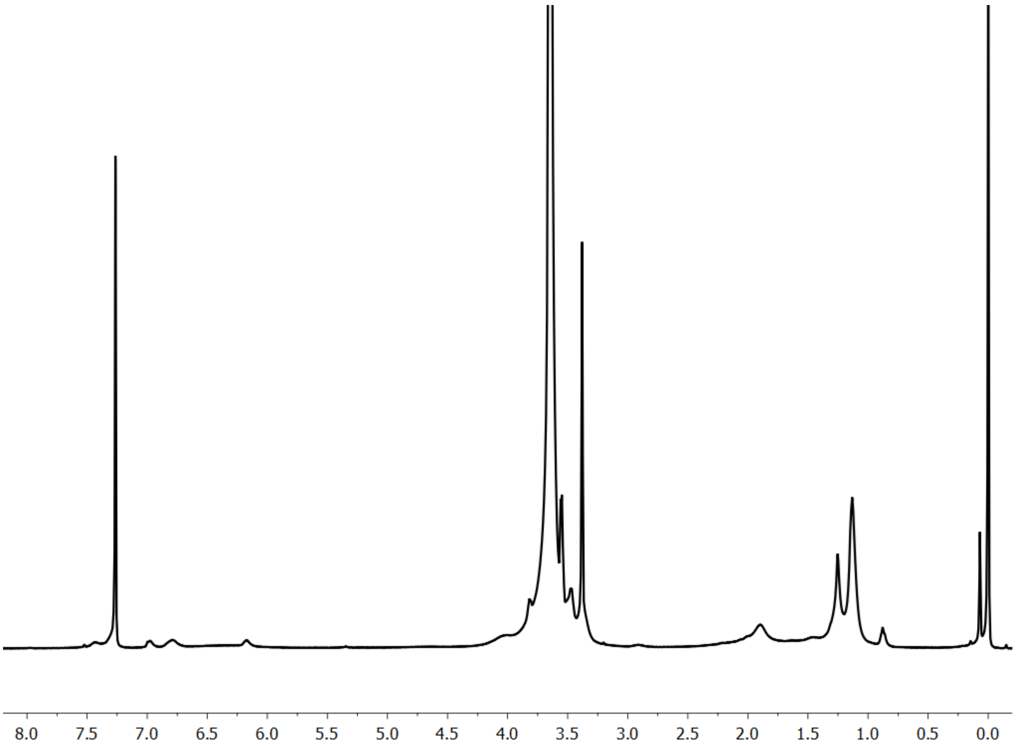

Figure S9: ^{1}H NMR spectrum of polymer **P2** (400 MHz, $CDCl_3$, 25 °C). Signals for the incorporated PTH catalyst are visible in the aromatic region between 6.0–7.5 ppm.

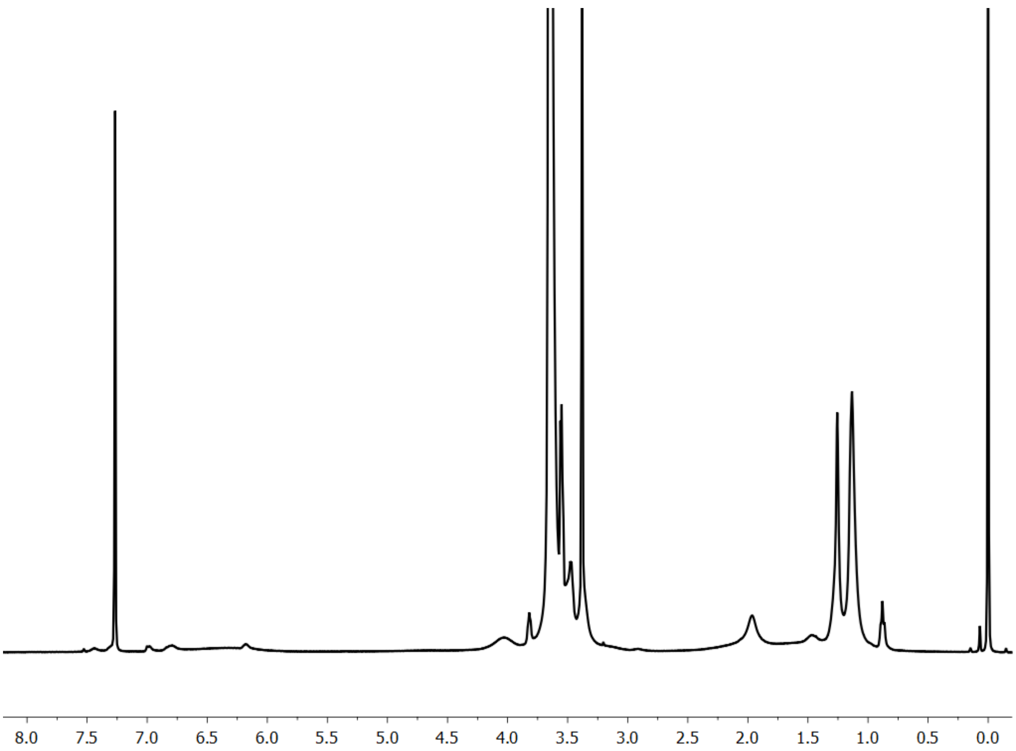

Figure S10: ^{1}H NMR spectrum of polymer **P3** (400 MHz, $CDCl_3$, 25 °C). Signals for the incorporated PTH catalyst are visible in the aromatic region between 6.0–7.5 ppm.

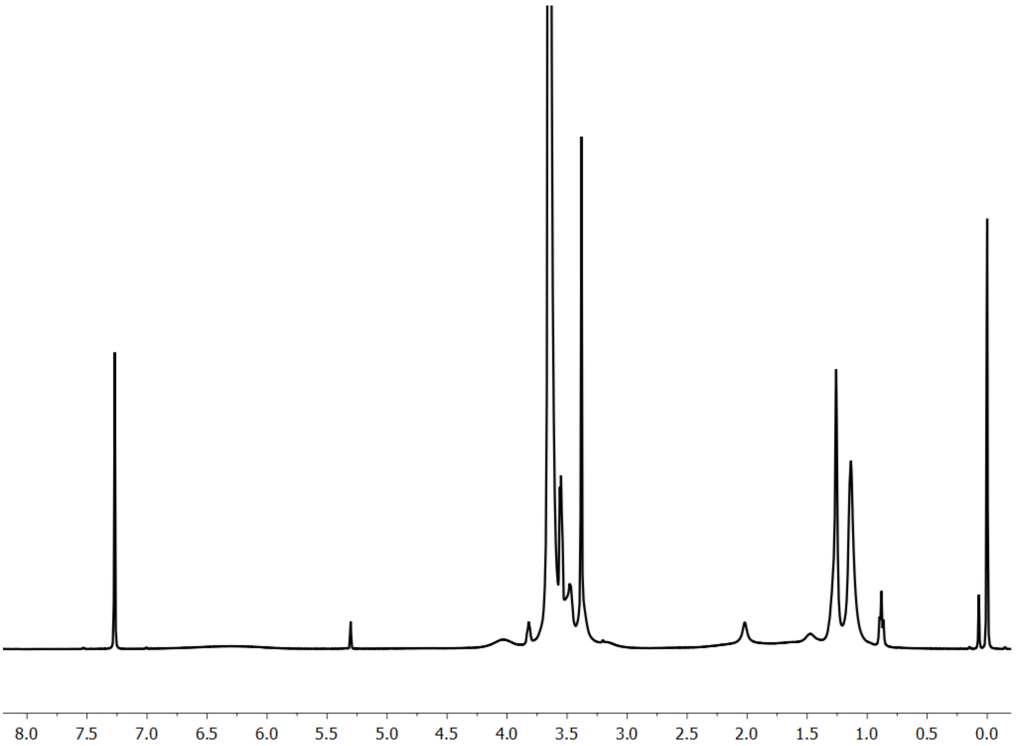

Figure S11: ^{1}H NMR spectrum of polymer **P4** (400 MHz, $CDCl_3$, 25 °C).

4. Photoredox catalysis experiments

Experimental protocol (optimized reaction conditions)

A 1.5 mL glass vial with septum screw cap was charged with Togni's reagent **1a** (30 µmol, 1.0 eq.), substrate (45 µmol, 1.5 eq.), and polymer **P3** (10 mg) and 0.8 mL of deionized water was added. After sealing the capped vial with parafilm and degassing the aqueous phase with argon for 5 min, the reaction mixture was illuminated with a focused 385 nm LED (irradiance E = 23 mW/cm^2) for 3 hours while being stirred and cooled with compressed air. Afterwards, trifluorotoluene (3.68 µL, 30 µmol, 1.0 eq.) as the internal ^{19}F NMR standard (δ = −62.75 ppm in $CDCl_3$) was added and the mixture was extracted three times with 0.5 mL of deuterated chloroform. Product formation was determined via quantitative ^{19}F NMR spectroscopy with trifluorotoluene (1.0 eq.) as the internal ^{19}F NMR standard (with respect to consumed **1a**) and verified by GC-MS if applicable.

Experiment with sunlight (details regarding Figure 5a)

Samples were prepared according to the experimental protocol with **2a** as the substrate. The experiment under sunlight irradiation was carried out on the 30th of July 2021 from 10 am to 5 pm (max. temperature 26 °C, partly cloudy). Product formation was determined via quantitative NMR spectroscopy with trifluorotoluene (1.0 eq.) as the internal ^{19}F NMR standard.

Recycling experiment (details regarding Figure 5b)

An aqueous mixture with 1,3,5-trimethoxybenzene as the substrate was prepared according to the experimental protocol. The mixture was first illuminated with a 385 nm LED for 3 h while being stirred continuously and cooled by a stream of compressed air to keep it at room temperature. The aqueous mixture was subsequently extracted 10x with 0.5 mL of diethylether. After removal of the organic solvent, the conversion was determined by ^{19}F NMR spectroscopy in $CDCl_3$. In the meantime, the vial with the aqueous phase was placed in a pre-heated oil bath at 50 °C for 1 h to remove residual diethylether. The same amounts of 1,3,5-trimethoxybenzene and Togni's reagent **1a** were subsequently added to the water solution and the procedure was repeated anew (in total three cycles).

Experiment with TEMPO (details regarding Figure 5c)

An aqueous mixture with *N*-phenylpyrrole **2a** as the substrate in addition to TEMPO (150 µmol, 23.4 mg, 5.0 eq.) was prepared according to the experimental protocol. The mixture was illuminated for 18 h and product formation was determined by quantitative ^{19}F NMR spectroscopy with trifluorotoluene (1.0 eq.) as the internal ^{19}F NMR standard (see Figure S33).

Experiment with α-methylstyrene (details regarding Figure 5d)

An aqueous mixture with α-methylstyrene **5a** as the substrate (90 µmol, 11.7 µL, 3.0 eq.) was prepared according to the experimental protocol. The mixture was illuminated for 7 h and product formation was determined by quantitative ^{19}F NMR spectroscopy with trifluorotoluene (1.0 eq.) as the internal ^{19}F NMR standard (see Figure S34).

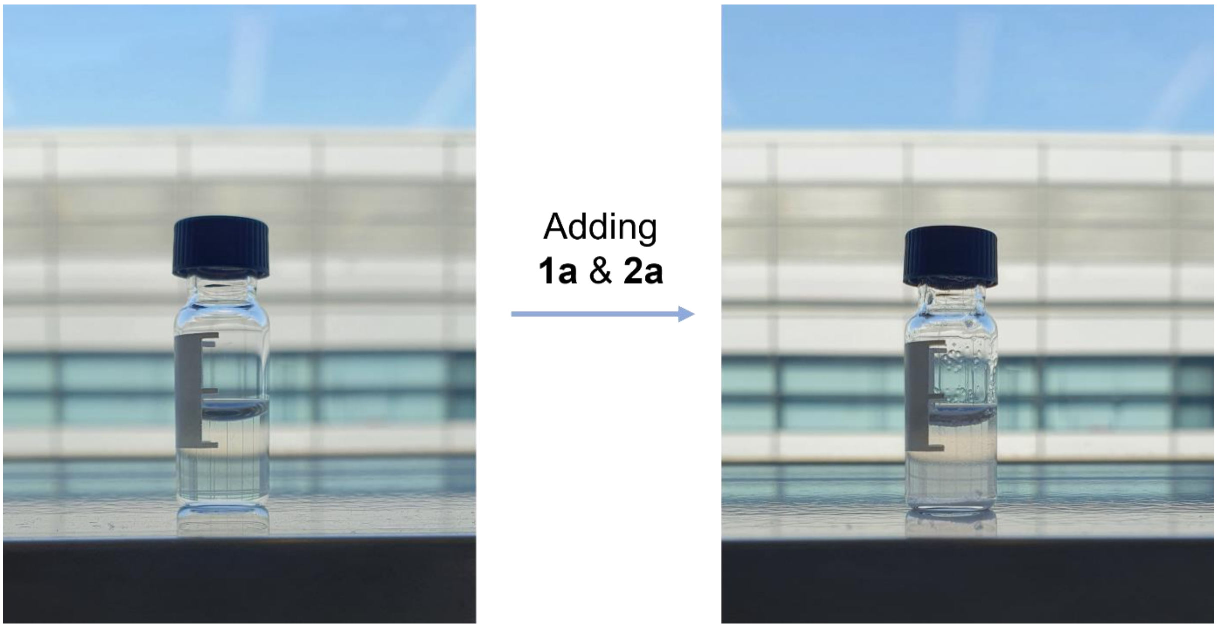

Figure S12: Photographs show a homogenous solution of **P3** (10 mg) in 0.8 mL of deionized water (left) and a heterogenous mixture after the addition of hydrophobic substrates **1a** and **2a** (right). The uptake of the reagents into the nanoparticles is driven by hydrophobic interactions. A constant exchange of hydrophobic compounds between the nanoparticles and the aqueous phase takes place.

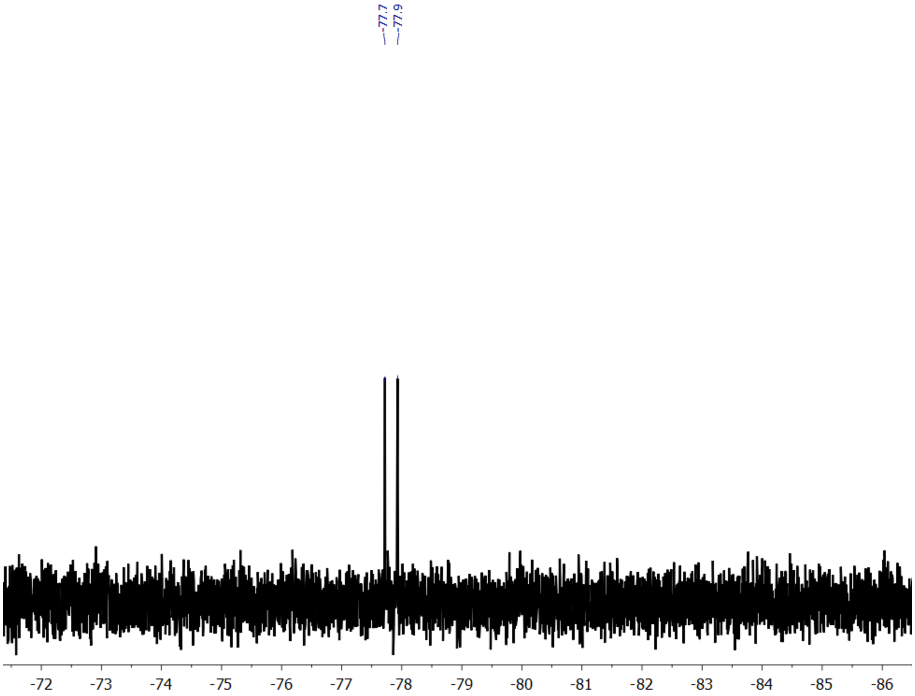

Figure S13: ^{19}F NMR spectrum showing traces of CF_3H ($J_{H,F}$ = 79.5 Hz) as a side product after extracting the reaction mixture with $CDCl_3$ (375 MHz, 20 °C).

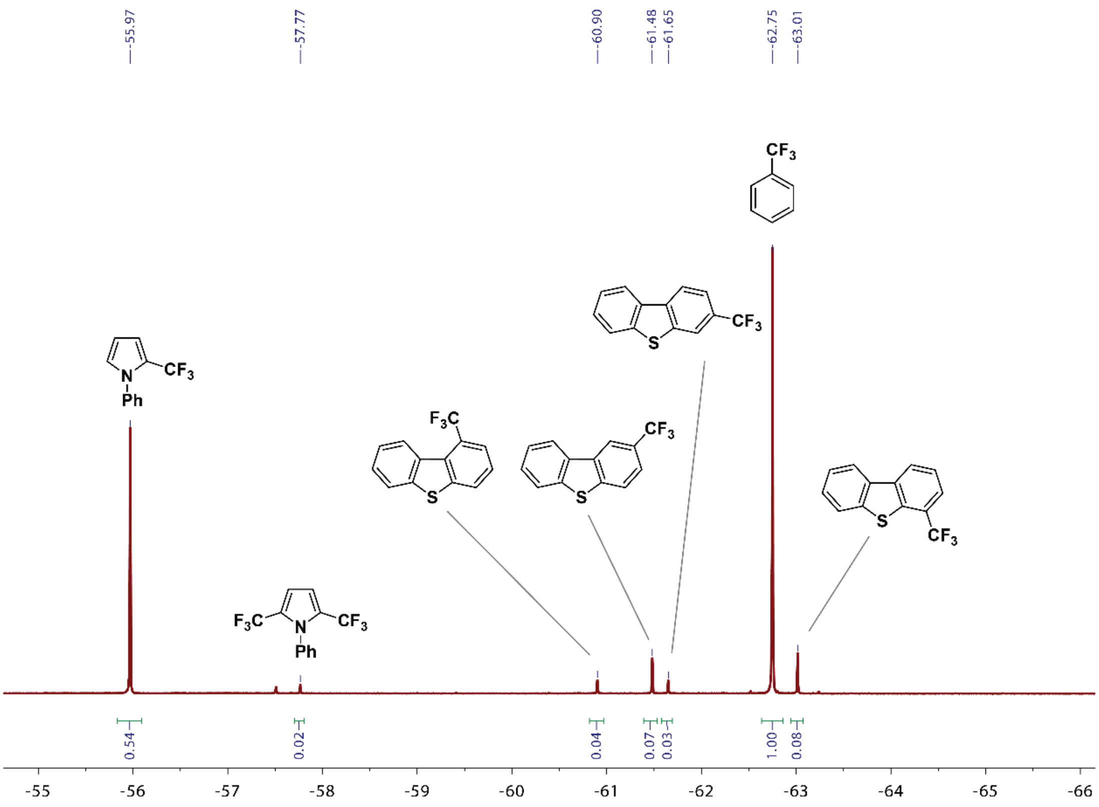

Figure S14: ^{19}F NMR spectrum in $CDCl_3$ showing the formation of trifluoromethylated dibenzothiophene side products by using the CF_3 reagent **1c**. The chemical shifts of the side products are in line with literature.[2]

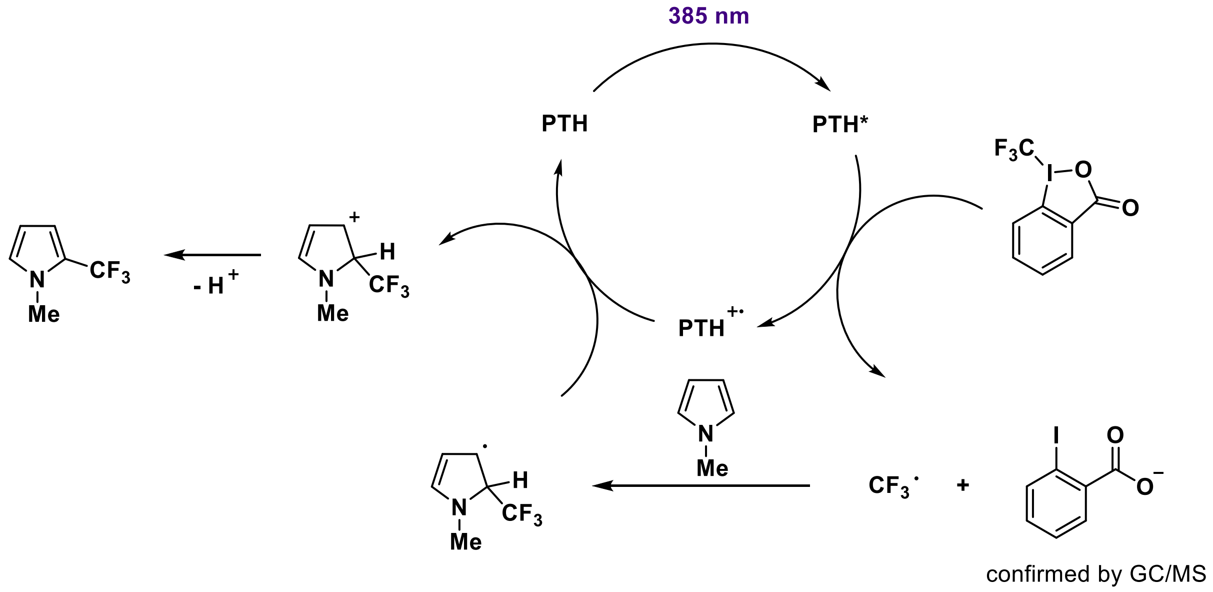

Figure S15: Possible reaction mechanism.

Major product: **1-Phenyl-2-(trifluoromethyl)-1*H*-pyrrole (2b)**

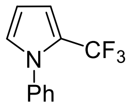

^{19}F NMR (376 MHz, $CDCl_3$, 25 °C): δ = −56.0 (s, 3F, CF_3) ppm. (literature: −56.0 ppm)[3]

GC-MS-EI (m/z) calc. for $C_{11}H_8F_3N$ $[M]^+$: 211, found 211.

Minor product: **1-Phenyl-2,5-bis(trifluoromethyl)-1*H*-pyrrole (2c)**

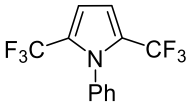

^{19}F NMR (376 MHz, $CDCl_3$, 25 °C): δ = −57.8 (s, 3F, CF_3) ppm.

GC-MS-EI (m/z) calc. for $C_{12}H_8F_6N$ $[M]^+$: 279, found 279.

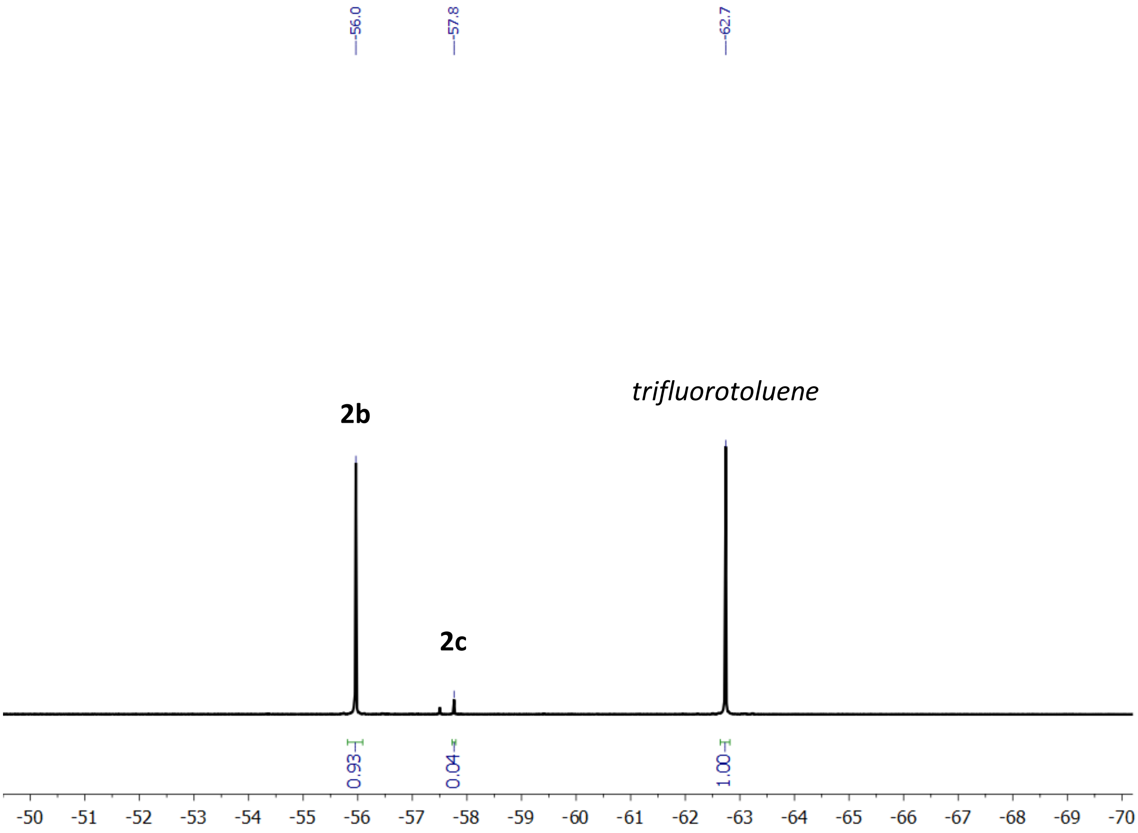

Figure S16: ^{19}F NMR spectrum in $CDCl_3$ after extraction of the aqueous reaction mixture with $CDCl_3$ to determine the product formation with trifluorotoluene (1 eq.) as internal standard.

Major product: **1-Methyl-2-(trifluoromethyl)-1*H*-pyrrole (3a)**

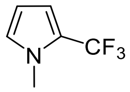

^{19}F NMR (376 MHz, $CDCl_3$, 25 °C): δ = −58.8 (s, 3F, CF_3) ppm. (literature: −58.8 ppm)[4,5]

GC-MS-EI (m/z) calc. for $C_6H_5F_3N$ $[M]^+$: 149, found 149.

Minor product: **1-Methyl-2,5-bis(trifluoromethyl)-1*H*-pyrrole (3a*)**

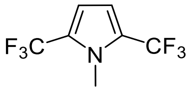

^{19}F NMR (376 MHz, $CDCl_3$, 25 °C): δ = −59.6 (s, 3F, CF_3) ppm. (literature: −60.2 ppm)[6]

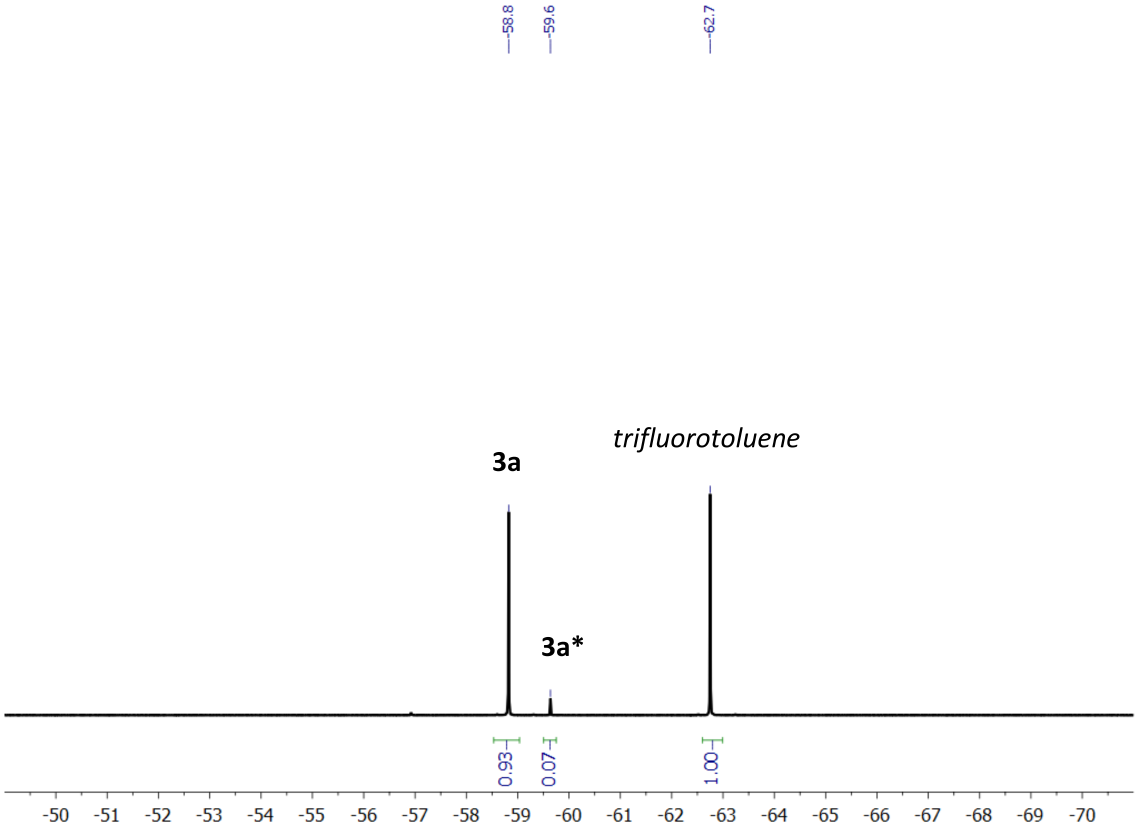

Figure S17: ^{19}F NMR spectrum in $CDCl_3$ after extraction of the aqueous reaction mixture with $CDCl_3$ to determine the product formation with trifluorotoluene (1 eq.) as internal standard.

Major product: **2-(Trifluoromethyl)-1*H*-pyrrole (3b)**

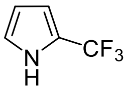

^{19}F NMR (376 MHz, $CDCl_3$, 25 °C): δ = −59.2 (s, 3F, CF_3) ppm. (literature: −59.0 ppm)[7]

GC-MS-EI (m/z) calc. for $C_5H_4F_3N$ $[M]^+$: 135, found 135.

Minor product: **2-(Trifluoromethyl)-1*H*-pyrrole (3b*)**

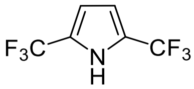

^{19}F NMR (376 MHz, $CDCl_3$, 25 °C): δ = −60.1 (s, 3F, CF_3) ppm. (literature: −61.2 ppm)[17]

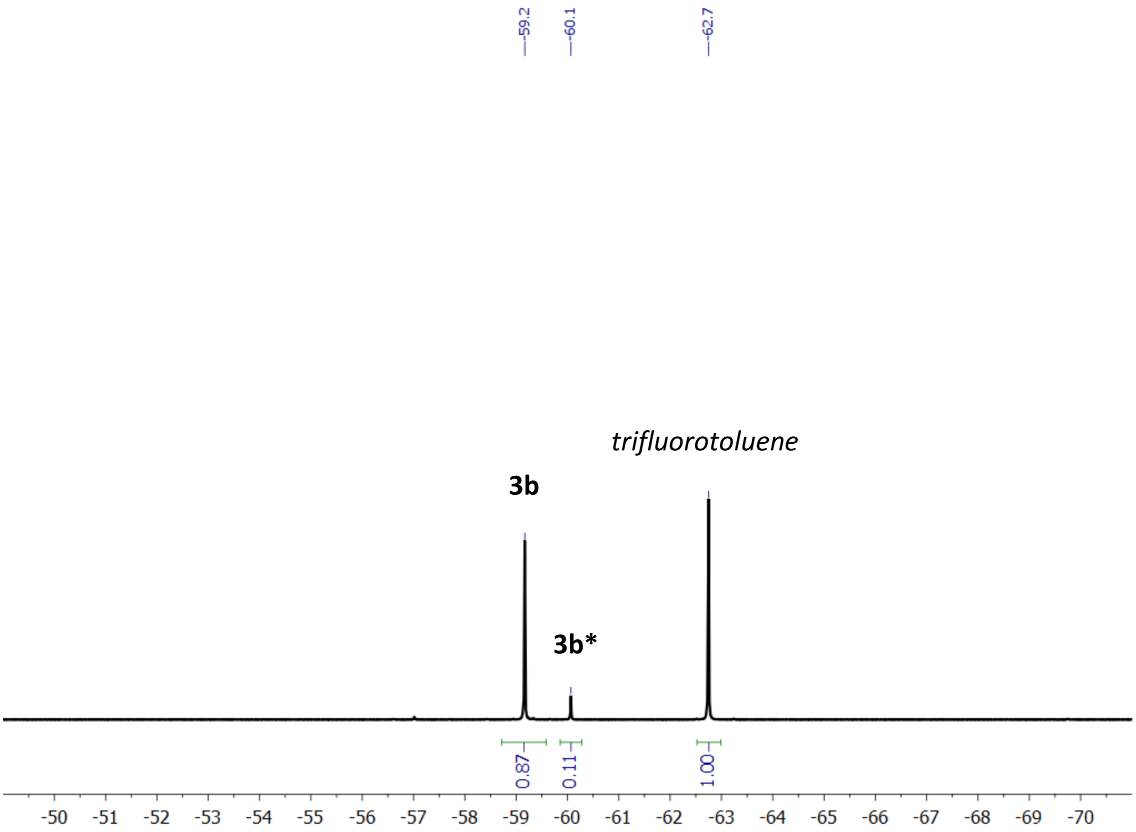

Figure S18: ^{19}F NMR spectrum in $CDCl_3$ after extraction of the aqueous reaction mixture with $CDCl_3$ to determine the product formation with trifluorotoluene (1 eq.) as internal standard.

1-(1-Methyl-5-(trifluoromethyl)-1*H*-pyrrol-2-yl)ethan-1-one (3c)

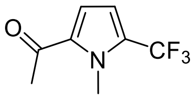

^{19}F NMR (376 MHz, $CDCl_3$, 25 °C): δ = −59.8 (s, 3F, CF_3) ppm. (literature: −59.8 ppm)[5]

GC-MS-EI (m/z) calc. for $C_8H_8F_3NO$ $[M]^+$: 191, found 191.

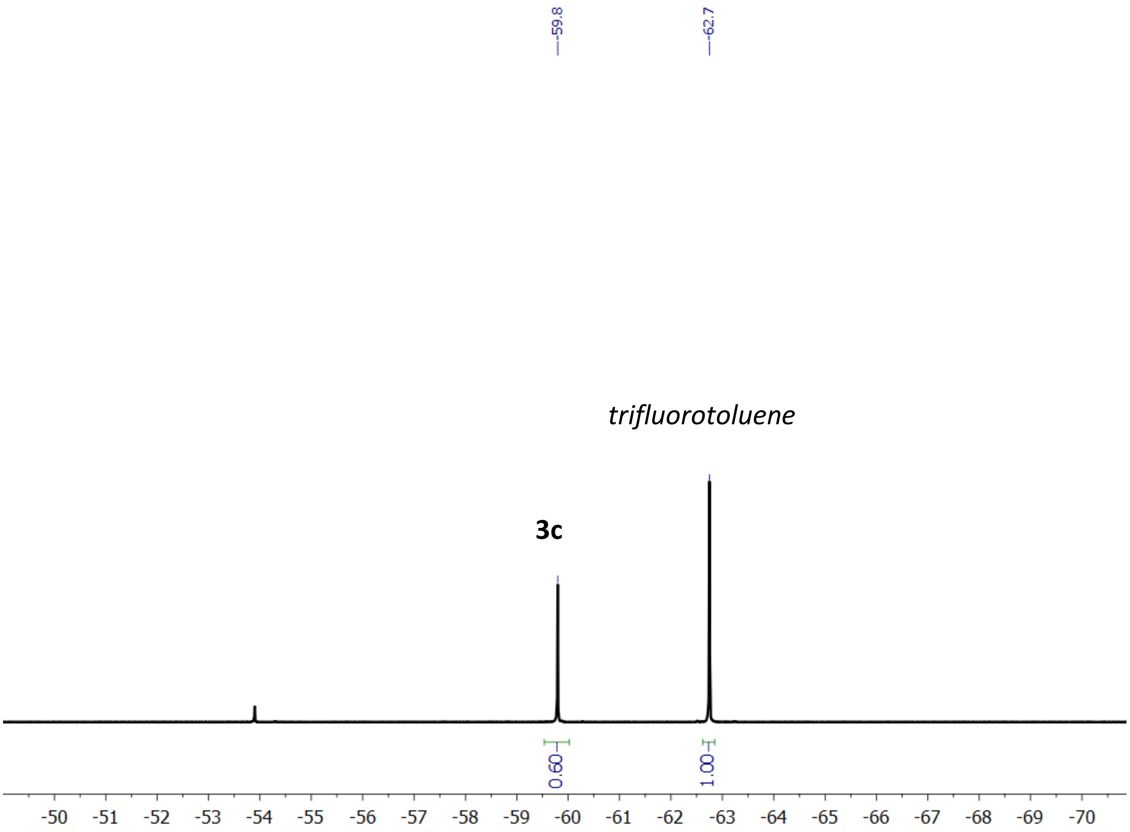

Figure S19: ^{19}F NMR spectrum in $CDCl_3$ after extraction of the aqueous reaction mixture with $CDCl_3$ to determine the product formation with trifluorotoluene (1 eq.) as internal standard.

tert-Butyl 2-(trifluoromethyl)-1H-pyrrole-1-carboxylate (3d)

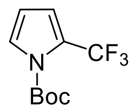

^{19}F NMR (376 MHz, $CDCl_3$, 25 °C): δ = −58.3 (s, 3F, CF_3) ppm. (literature: −58.3 ppm)[5,8]

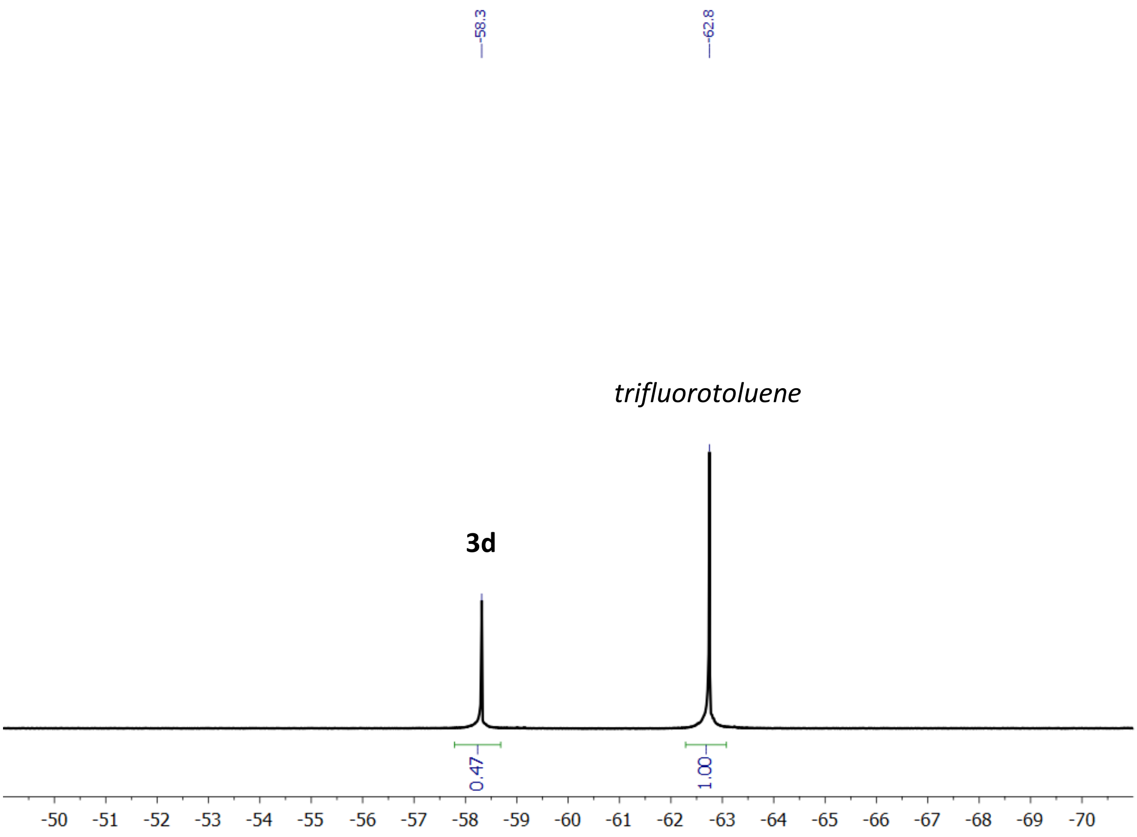

Figure S20: ^{19}F NMR spectrum in $CDCl_3$ after extraction of the aqueous reaction mixture with $CDCl_3$ to determine the product formation with trifluorotoluene (1 eq.) as internal standard.

1-Benzyl-2-(trifluoromethyl)-1*H*-pyrrole (3e)

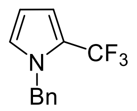

^{19}F NMR (376 MHz, $CDCl_3$, 25 °C): δ = −57.7 (s, 3F, CF_3) ppm. (literature: −57.5 ppm)[18]

GC-MS-EI (m/z) calc. for $C_{12}H_{10}F_3N$ $[M]^+$: 225, found 225.

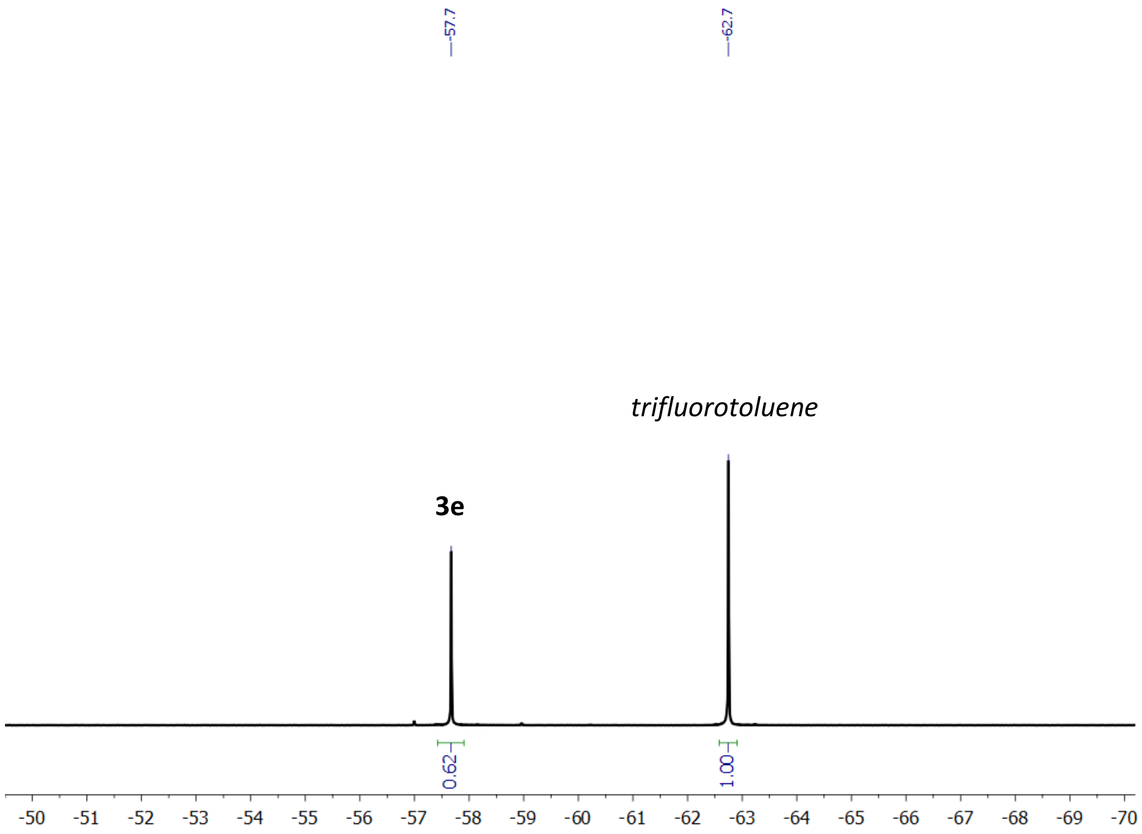

Figure S21: ^{19}F NMR spectrum in $CDCl_3$ after extraction of the aqueous reaction mixture with $CDCl_3$ to determine the product formation with trifluorotoluene (1 eq.) as internal standard.

Major product: **1-(4-Chlorophenyl)-2-(trifluoromethyl)-1*H*-pyrrole (3f)**

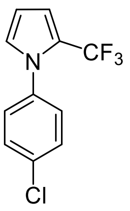

^{19}F NMR (376 MHz, $CDCl_3$, 25 °C): δ = −56.0 (s, 3F, CF_3) ppm. (literature: −56.4 ppm)[9]

GC-MS-EI (m/z) calc. for $C_{11}H_7ClF_3N$ $[M]^+$: 245, found 245.

Minor product: **1-(4-chlorophenyl)-2,5-bis(trifluoromethyl)-1*H*-pyrrole (3f*)**

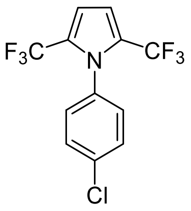

^{19}F NMR (376 MHz, $CDCl_3$, 25 °C): δ = −57.7 (s, 6F, CF_3) ppm.

GC-MS-EI (m/z) calc. for $C_{12}H_6ClF_6N$ $[M]^+$: 313, found 313.

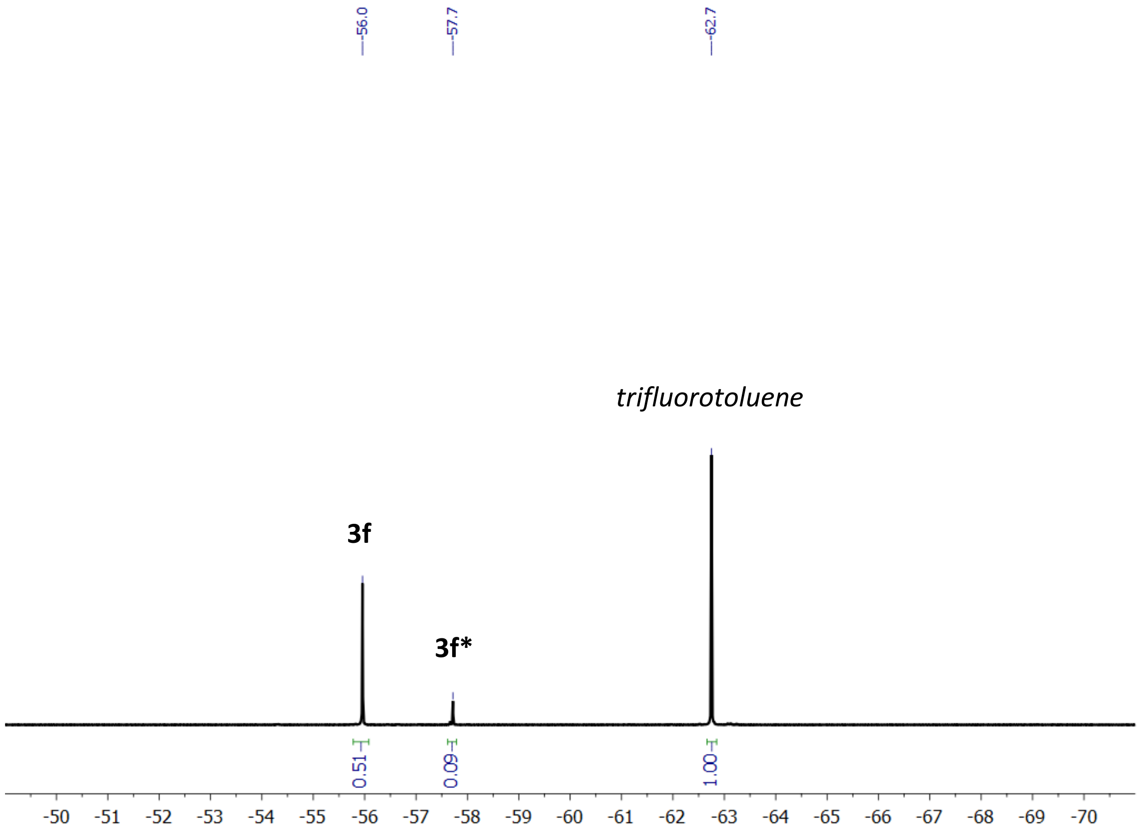

Figure S22: ^{19}F NMR spectrum in $CDCl_3$ after extraction of the aqueous reaction mixture with $CDCl_3$ to determine the product formation with trifluorotoluene (1 eq.) as internal standard.

1-Methyl-2-(trifluoromethyl)-1*H*-indole (3g)

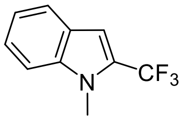

^{19}F NMR (376 MHz, $CDCl_3$, 25 °C): δ = −59.6 (s, 3F, CF_3) ppm. (literature: −59.5 ppm)[10]

GC-MS-EI (m/z) calc. for $C_{10}H_8F_3N$ $[M]^+$: 199, found 199.

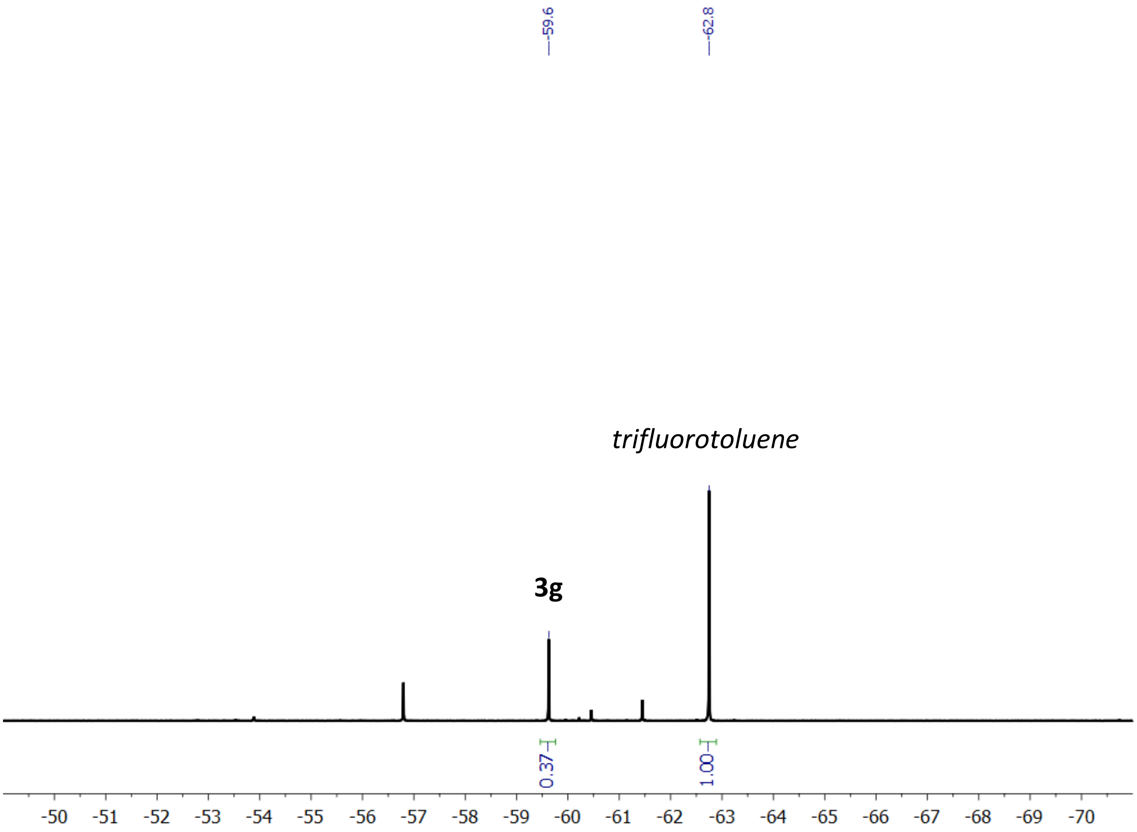

Figure S23: ^{19}F NMR spectrum in $CDCl_3$ after extraction of the aqueous reaction mixture with $CDCl_3$ to determine the product formation with trifluorotoluene (1 eq.) as internal standard.

3-Methyl-2-(trifluoromethyl)-1*H*-indole (3h)

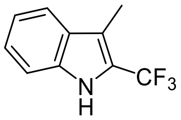

^{19}F NMR (376 MHz, $CDCl_3$, 25 °C): δ = −58.6 (s, 3F, CF_3) ppm. (literature: −58.6 ppm)[10]

GC-MS-EI (m/z) calc. for $C_{10}H_8F_3N$ $[M]^+$: 199, found 199.

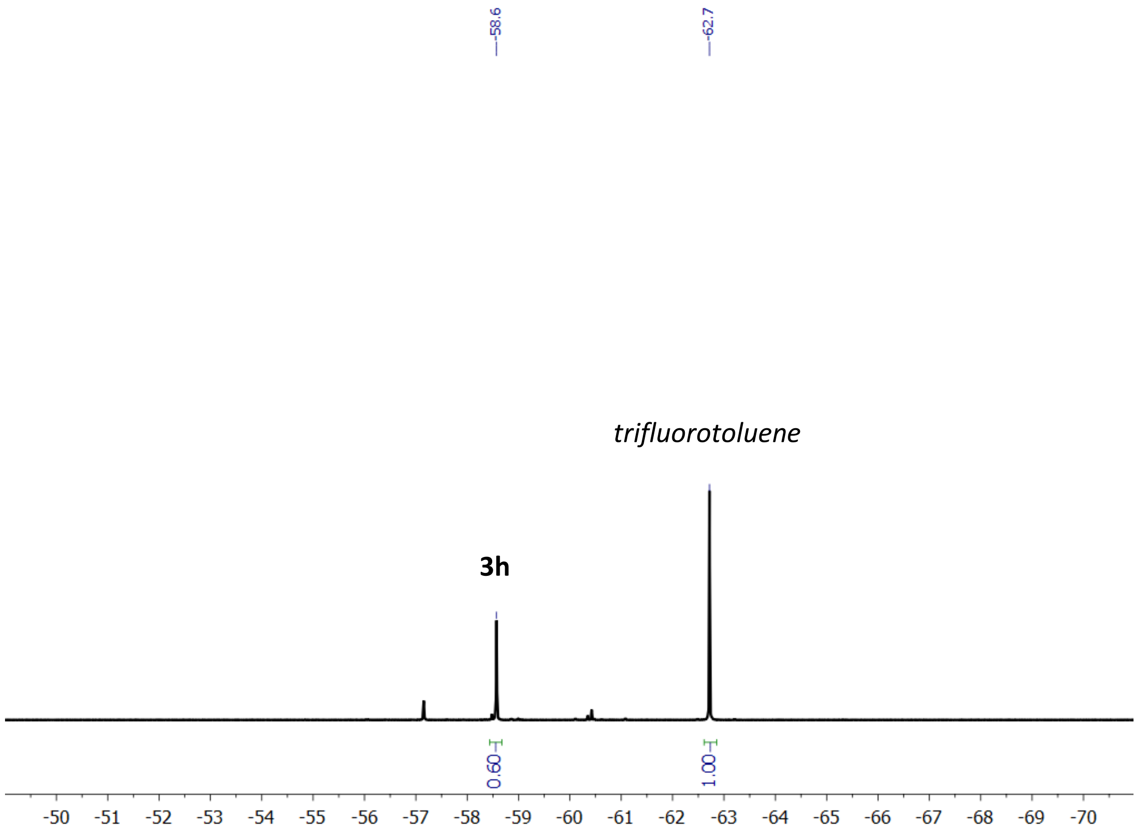

Figure S24: ^{19}F NMR spectrum in $CDCl_3$ after extraction of the aqueous reaction mixture with $CDCl_3$ to determine the product formation with trifluorotoluene (1 eq.) as internal standard.

Ethyl 2-acetamido-3-(2-(trifluoromethyl)-1*H*-indol-3-yl)propanoate (3i)

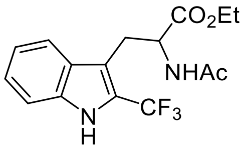

^{19}F NMR (376 MHz, $CDCl_3$, 25 °C): δ = −57.8 (s, 3F, CF_3) ppm. (literature: −57.9 ppm)[11]

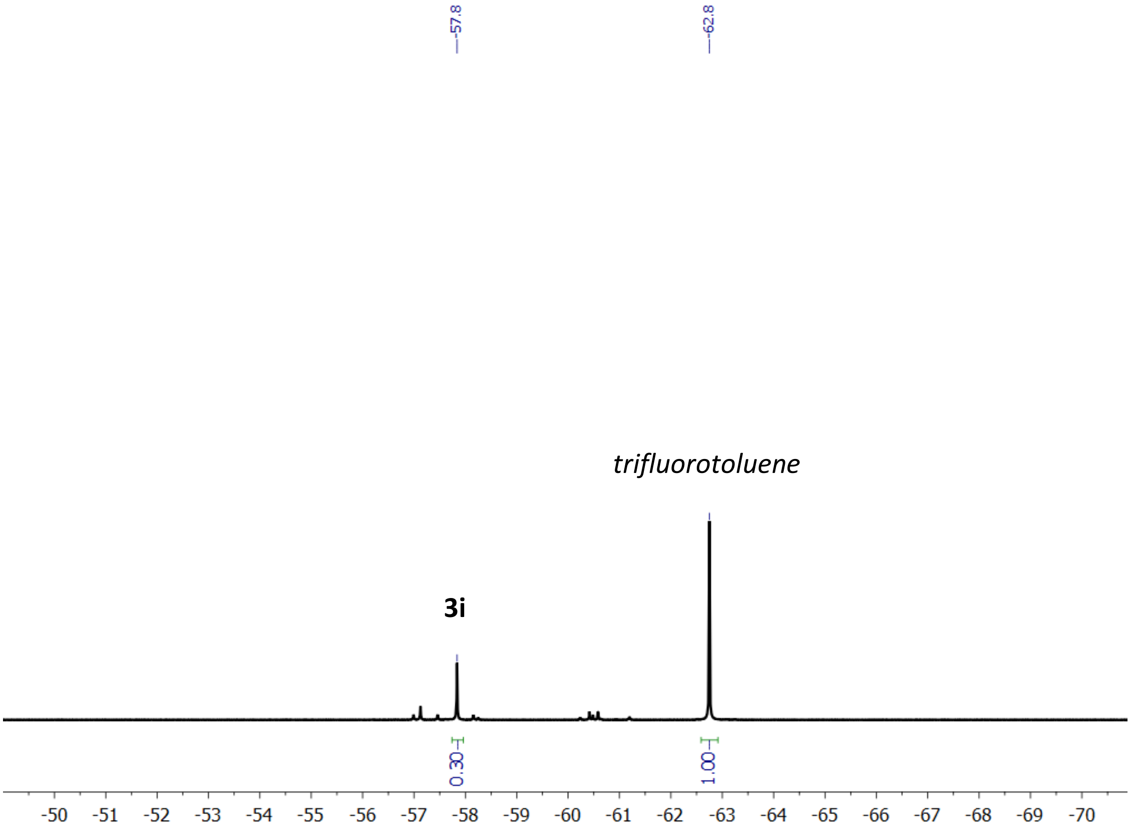

Figure S25: ^{19}F NMR spectrum in $CDCl_3$ after extraction of the aqueous reaction mixture with $CDCl_3$ to determine the product formation with trifluorotoluene (1 eq.) as internal standard.

N-(2-(5-methoxy-2-(trifluoromethyl)-1H-indol-3-yl)ethyl)acetamide (3j)

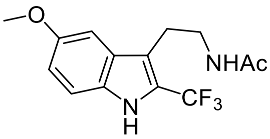

^{19}F NMR (376 MHz, $CDCl_3$, 25 °C): δ = −57.9 (s, 3F, CF_3) ppm. (literature: −57.8 ppm)[11]

GC-MS-EI (m/z) calc. for $C_{14}H_{15}F_3N_2O_2$ $[M]^+$: 300, found 300.

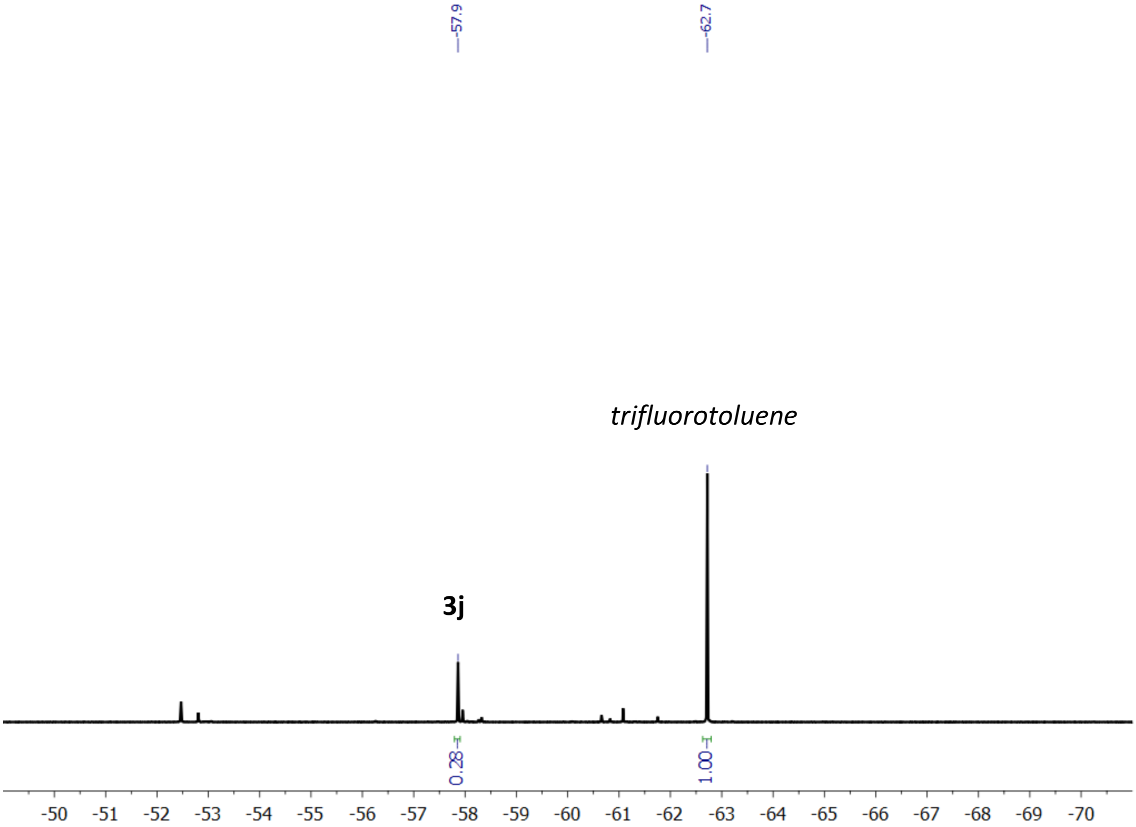

Figure S26: ^{19}F NMR spectrum in $CDCl_3$ after extraction of the aqueous reaction mixture with $CDCl_3$ to determine the product formation with trifluorotoluene (1 eq.) as internal standard.

1-Methyl-2-(trifluoromethyl)-1*H*-imidazole (3k)

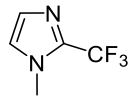

^{19}F NMR (376 MHz, $CDCl_3$, 25 °C): δ = −62.0 (s, 3F, CF_3) ppm. (literature: −62.0 ppm)[10]

GC-MS-EI (m/z) calc. for $C_5H_5F_3N_2$ $[M]^+$: 150, found 150.

1-Methyl-5-(trifluoromethyl)-1*H*-imidazole (3k°)

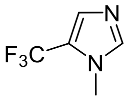

^{19}F NMR (376 MHz, $CDCl_3$, 25 °C): δ = −59.8 (s, 3F, CF_3) ppm. (literature: −59.7 ppm)[10]

GC-MS-EI (m/z) calc. for $C_5H_5F_3N_2$ $[M]^+$: 150, found 150.

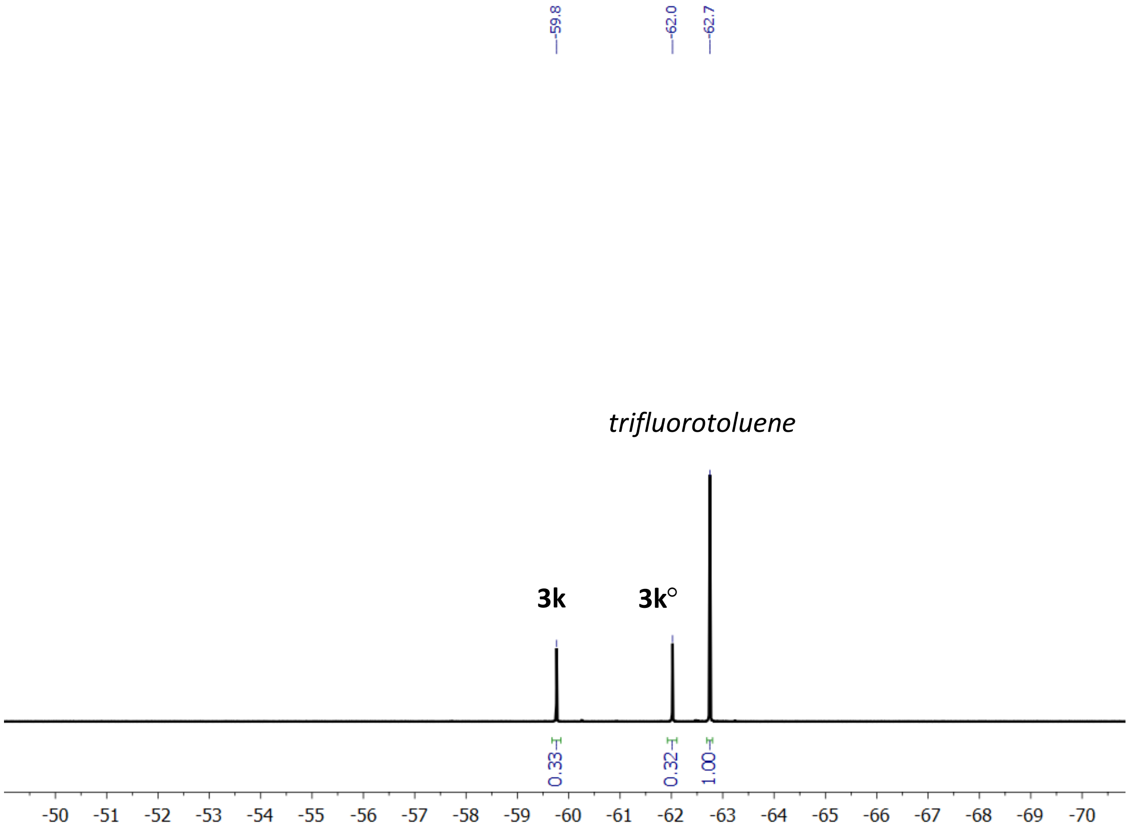

Figure S27: ^{19}F NMR spectrum in $CDCl_3$ after extraction of the aqueous reaction mixture with $CDCl_3$ to determine the product formation with trifluorotoluene (1 eq.) as internal standard.

1,3,7-Trimethyl-8-(trifluoromethyl)-3,7-dihydro-1*H*-purine-2,6-dione (3l)

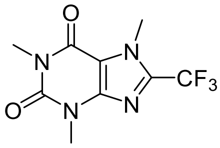

^{19}F NMR (376 MHz, $CDCl_3$, 25 °C): δ = −62.4 (s, 3F, CF_3) ppm. (literature: −62.4 ppm)[3]

GC-MS-EI (m/z) calc. for $C_9H_9F_3N_4O_2$ $[M]^+$: 262, found 262.

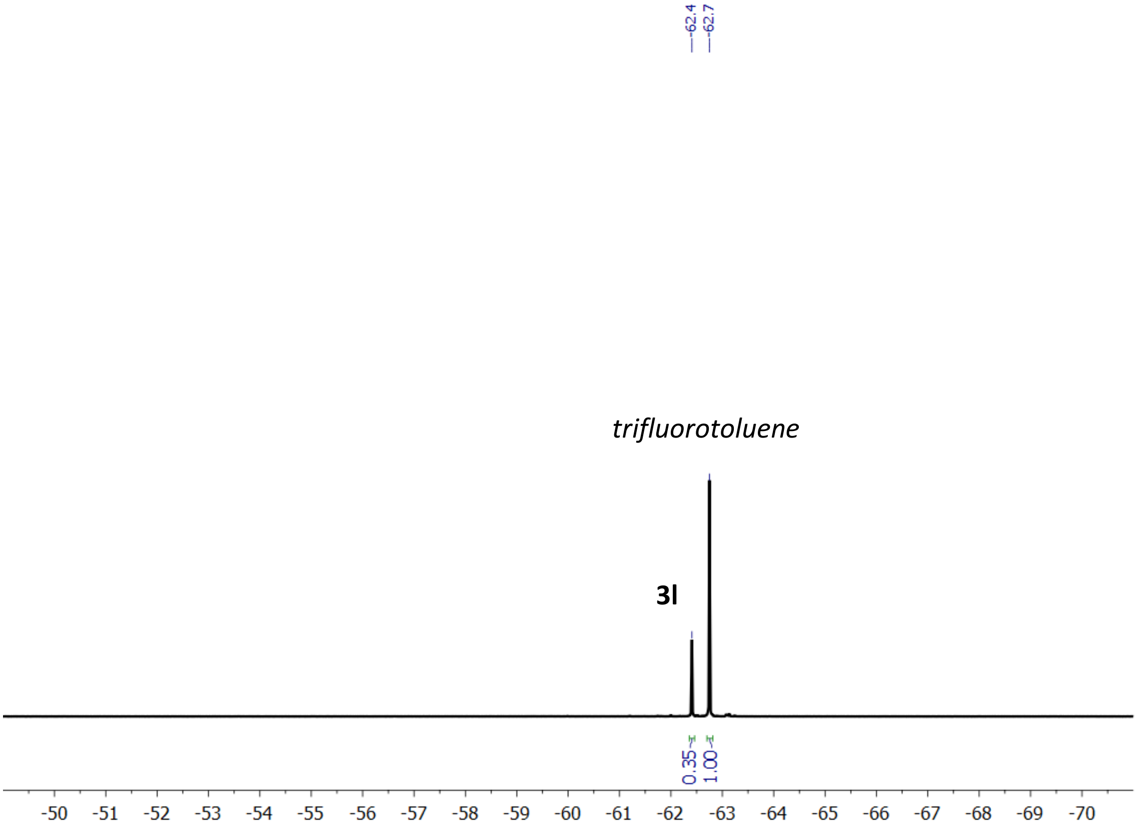

Figure S28: ^{19}F NMR spectrum in $CDCl_3$ after extraction of the aqueous reaction mixture with $CDCl_3$ to determine the product formation with trifluorotoluene (1 eq.) as internal standard.

1,4-Dimethoxy-2-(trifluoromethyl)benzene (3m)

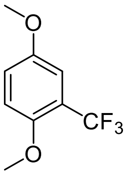

^{19}F NMR (376 MHz, $CDCl_3$, 25 °C): δ = −62.4 (s, 3F, CF_3) ppm. (literature: −62.4 ppm)[3–5,12]

GC-MS-EI (m/z) calc. for $C_9H_9F_3O_2$ $[M]^+$: 206, found 206.

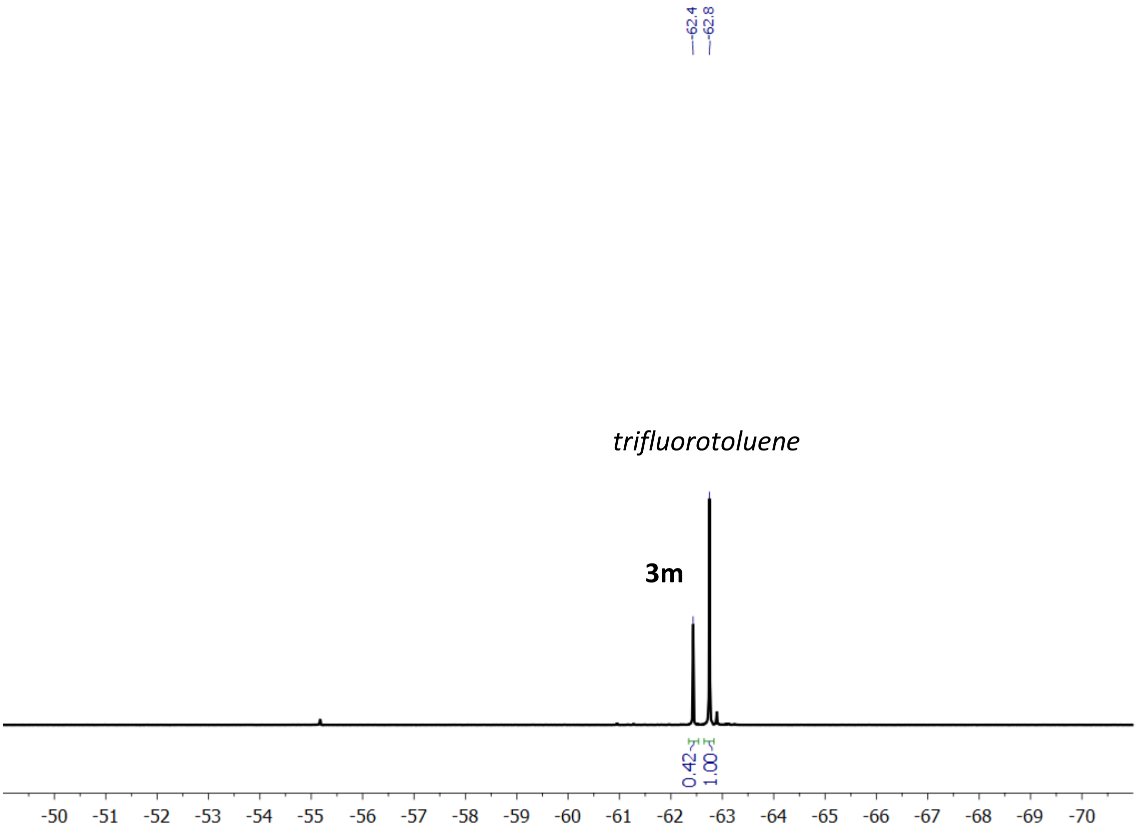

Figure S29: ^{19}F NMR spectrum in $CDCl_3$ after extraction of the aqueous reaction mixture with $CDCl_3$ to determine the product formation with trifluorotoluene (1 eq.) as internal standard.

3,4-Dimethoxy-5-(trifluoromethyl)benzaldehyde (3n)

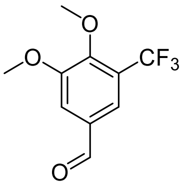

^{19}F NMR (376 MHz, $CDCl_3$, 25 °C): δ = −53.6 (s, 3F, CF_3) ppm. (literature: −54.0 ppm)[9]

GC-MS-EI (m/z) calc. for $C_{10}H_9F_3O_3$ $[M]^+$: 234, found 234.

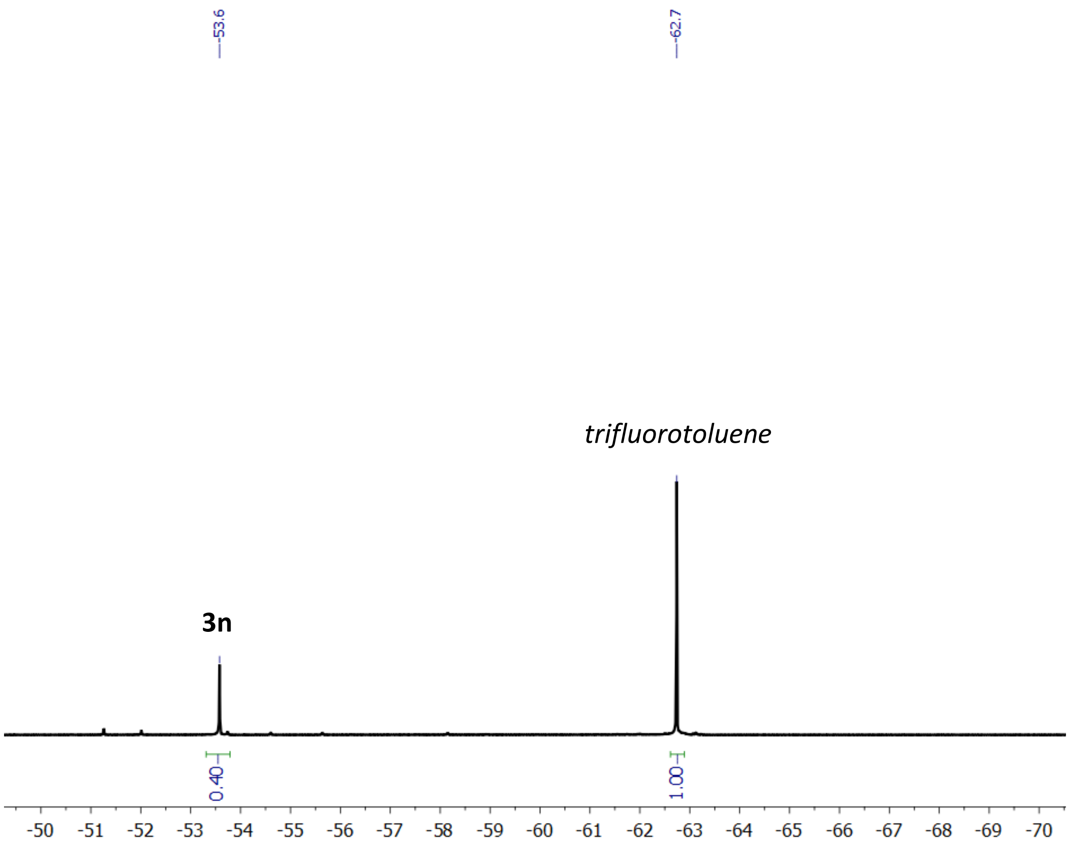

Figure S30: ^{19}F NMR spectrum in $CDCl_3$ after extraction of the aqueous reaction mixture with $CDCl_3$ to determine the product formation with trifluorotoluene (1 eq.) as internal standard.

Major Product: **1,2-Dimethoxy-4-(trifluoromethyl)benzene (3o)**

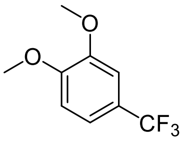

^{19}F NMR (376 MHz, $CDCl_3$, 25 °C): δ = −61.6 (s, 3F, CF_3) ppm. (literature: −61.6 ppm)[13]

GC-MS-EI (m/z) calc. for $C_9H_9F_3O_2$ $[M]^+$: 206, found 206.

Minor Product: **1,2-Dimethoxy-3-(trifluoromethyl)benzene (3o°)**

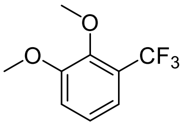

^{19}F NMR (376 MHz, $CDCl_3$, 25 °C): δ = −61.3 (s, 3F, CF_3) ppm. (literature: −61.3 ppm)[13]

GC-MS-EI (m/z) calc. for $C_9H_9F_3O_2$ $[M]^+$: 206, found 206.

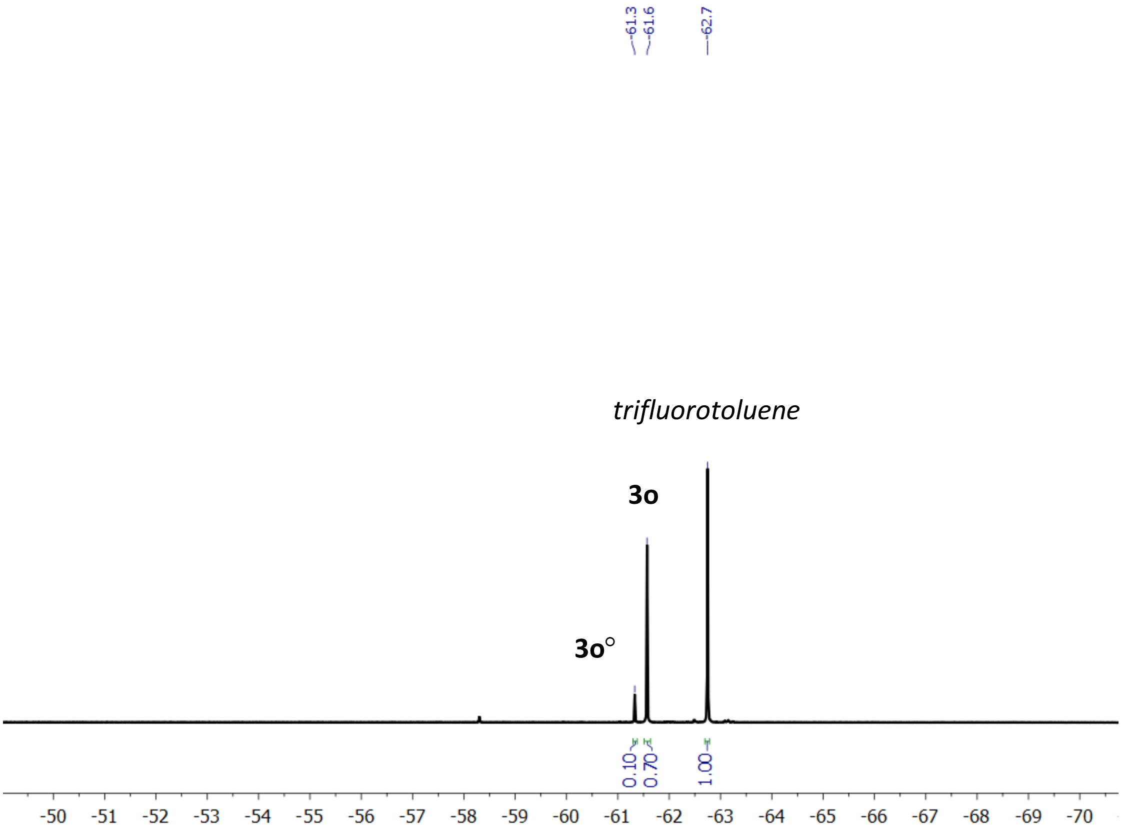

Figure S31: ^{19}F NMR spectrum in $CDCl_3$ after extraction of the aqueous reaction mixture with $CDCl_3$ to determine the product formation with trifluorotoluene (1 eq.) as internal standard.

Major Product: **1,3-Dimethoxy-4-(trifluoromethyl)benzene (3p)**

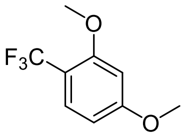

^{19}F NMR (376 MHz, $CDCl_3$, 25 °C): δ = −61.3 (s, 3F, CF_3) ppm. (literature: −61.3 ppm)[3,5]

GC-MS-EI (m/z) calc. for $C_9H_9F_3O_2$ $[M]^+$: 206, found 206.

Minor Product: **1,3-Dimethoxy-2-(trifluoromethyl)benzene (3p°)**

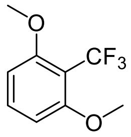

^{19}F NMR (376 MHz, $CDCl_3$, 25 °C): δ = −54.9 (s, 3F, CF_3) ppm. (literature: −54.9 ppm)[5]

GC-MS-EI (m/z) calc. for $C_9H_9F_3O_2$ $[M]^+$: 206, found 206.

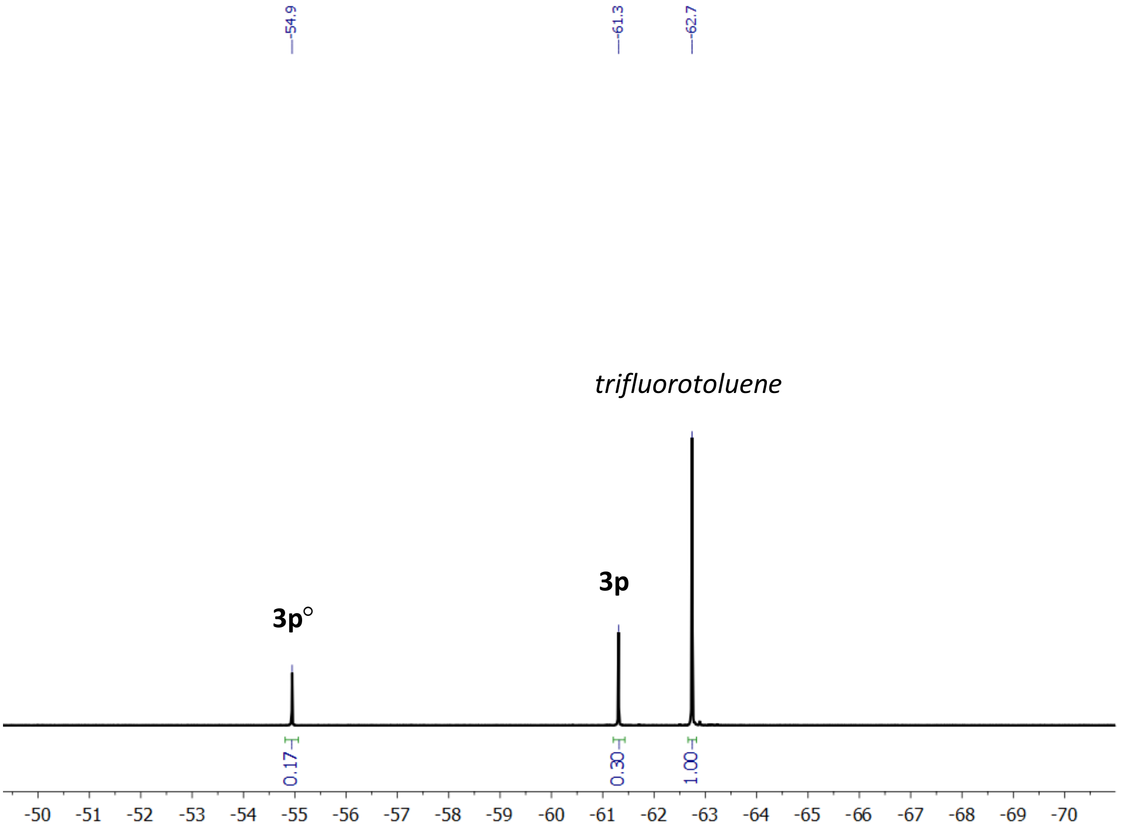

Figure S32: ^{19}F NMR spectrum in $CDCl_3$ after extraction of the aqueous reaction mixture with $CDCl_3$ to determine the product formation with trifluorotoluene (1 eq.) as internal standard.

Major Product: **1,3,5-Trimethoxy-2-(trifluoromethyl)benzene (3q)**

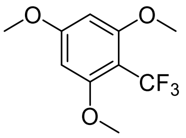

^{19}F NMR (376 MHz, $CDCl_3$, 25 °C): δ = −54.2 (s, 3F, CF_3) ppm. (literature: −54.2 ppm)[12]

GC-MS-EI (m/z) calc. for $C_{10}H_{11}F_3O_3$ $[M]^+$: 236, found 236.

Minor Product: **1,3,5-Trimethoxy-2,4-bis(trifluoromethyl)benzene (3q*)**

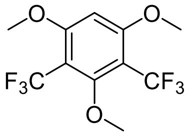

^{19}F NMR (376 MHz, $CDCl_3$, 25 °C): δ = −55.5 (s, 3F, CF_3) ppm. (literature: −55.5 ppm)[3]

GC-MS-EI (m/z) calc. for $C_{11}H_{11}F_6O_3$ $[M]^+$: 304, found 304.

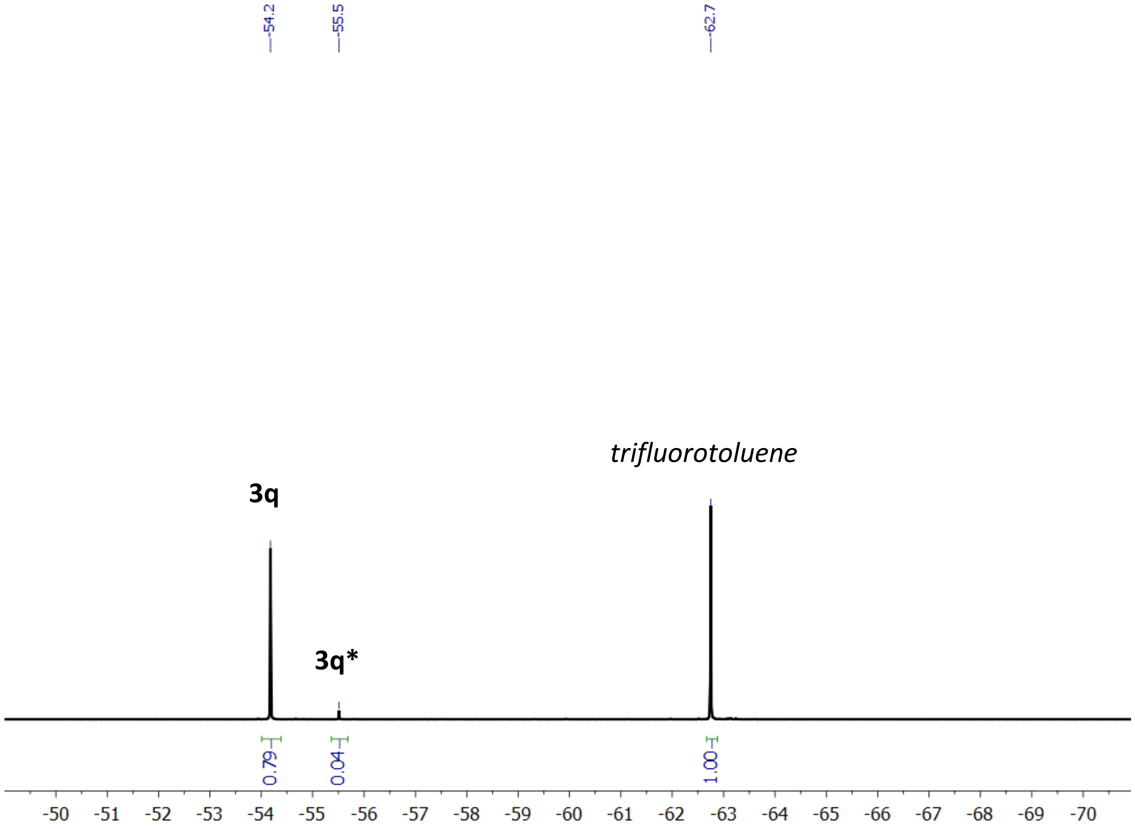

Figure S33: ^{19}F NMR spectrum in $CDCl_3$ after extraction of the aqueous reaction mixture with $CDCl_3$ to determine the product formation with trifluorotoluene (1 eq.) as internal standard.

1,2,3-Trimethoxy-5-methyl-4-(trifluoromethyl)benzene (3r)

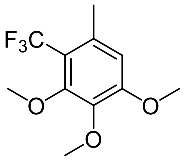

^{19}F NMR (376 MHz, $CDCl_3$, 25 °C): δ = −54.3 (s, 3F, CF_3) ppm. (literature: −54.3 ppm)[8]

GC-MS-EI (m/z) calc. for $C_{11}H_{13}F_3O_3$ $[M]^+$: 250, found 250.

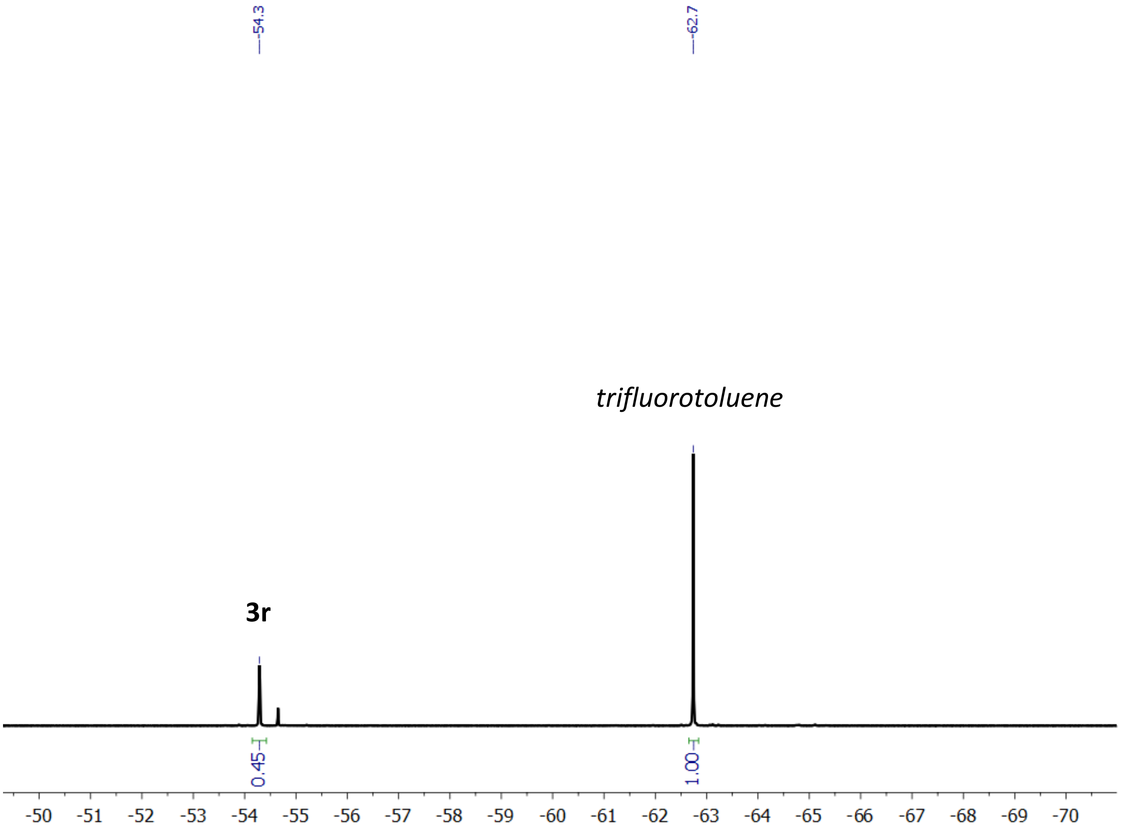

Figure S34: ^{19}F NMR spectrum in $CDCl_3$ after extraction of the aqueous reaction mixture with $CDCl_3$ to determine the product formation with trifluorotoluene (1 eq.) as internal standard.

2,2,6,6-Tetramethyl-1-(trifluoromethoxy)piperidine (4b)

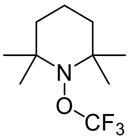

^{19}F NMR (376 MHz, $CDCl_3$, 25 °C): δ = −55.7 (s, 3F, CF_3) ppm. (literature: −55.7 ppm)[14]

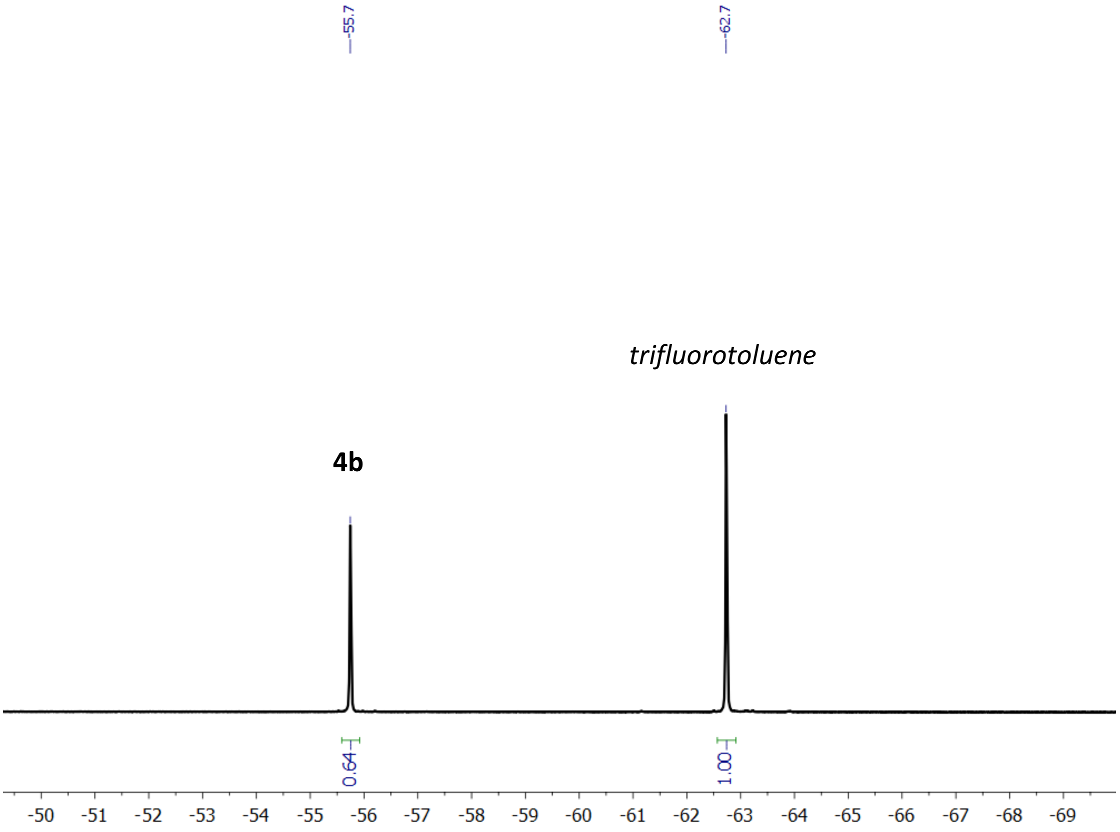

Figure S35: ^{19}F NMR spectrum in $CDCl_3$ after extraction of the aqueous reaction mixture with $CDCl_3$ to determine the product formation with trifluorotoluene (1 eq.) as internal standard.

Major product: **4,4,4-Trifluoro-2-phenylbutan-2-ol (5b)**

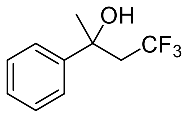

GC-MS-EI (m/z) calc. for $C_{10}H_9F_3$ $[M\text{-}H_2O]^+$: 186, found 186.

^{19}F-NMR (376 MHz, $CDCl_3$, 25 °C): δ = −60.1 ppm (t, $^3J_{F,H}$ = 10.9 Hz, 3F, CF_3). (literature: −60.1 ppm)[15]

Major product: **(4,4,4-Trifluorobut-1-en-2-yl)benzene**

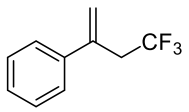

^{19}F NMR (376 MHz, $CDCl_3$, 25 °C): δ = −64.5 ppm (t, $^3J_{F,H}$ = 10.4 Hz, 3F, CF_3). (literature: −64.4 ppm)[16]

GC-MS-EI (m/z) calc. for $C_{10}H_9F_3$ $[M]^+$: 186, found 186.

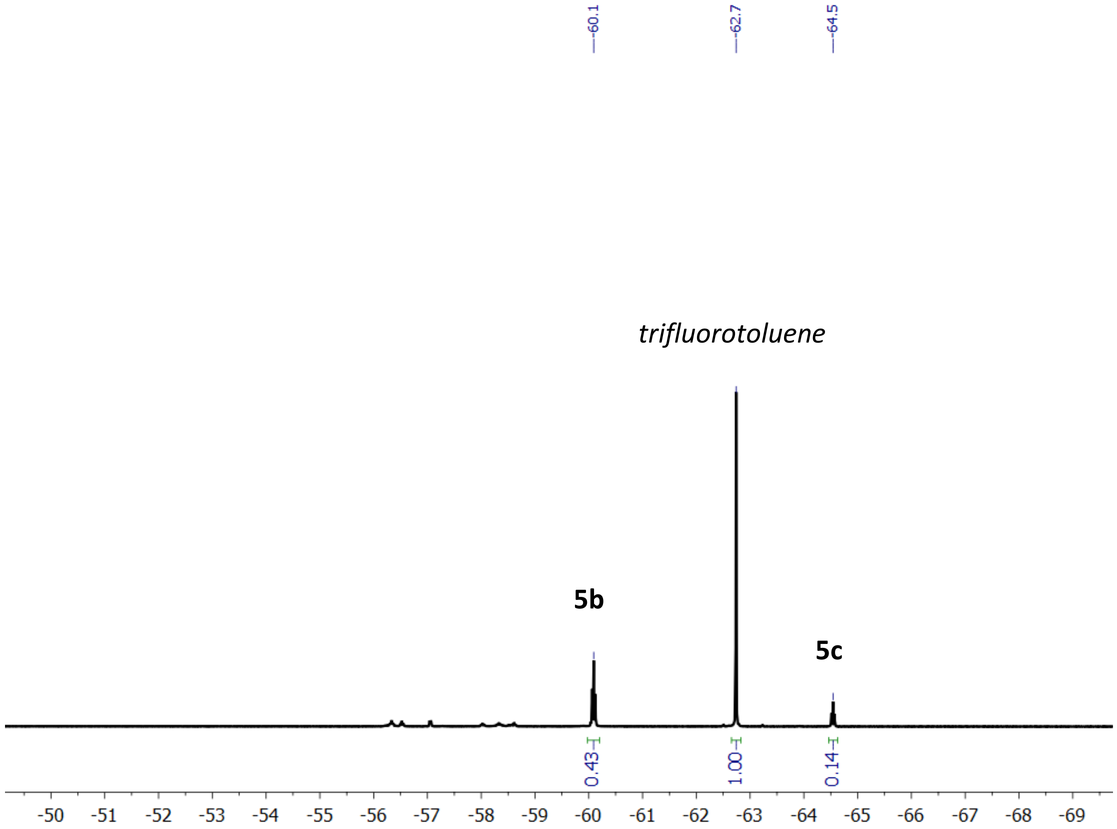

Figure S36: ^{19}F NMR spectrum in $CDCl_3$ after extraction of the aqueous reaction mixture with $CDCl_3$ to determine the product formation with trifluorotoluene (1 eq.) as internal standard.

5. NMR spectra of final compounds

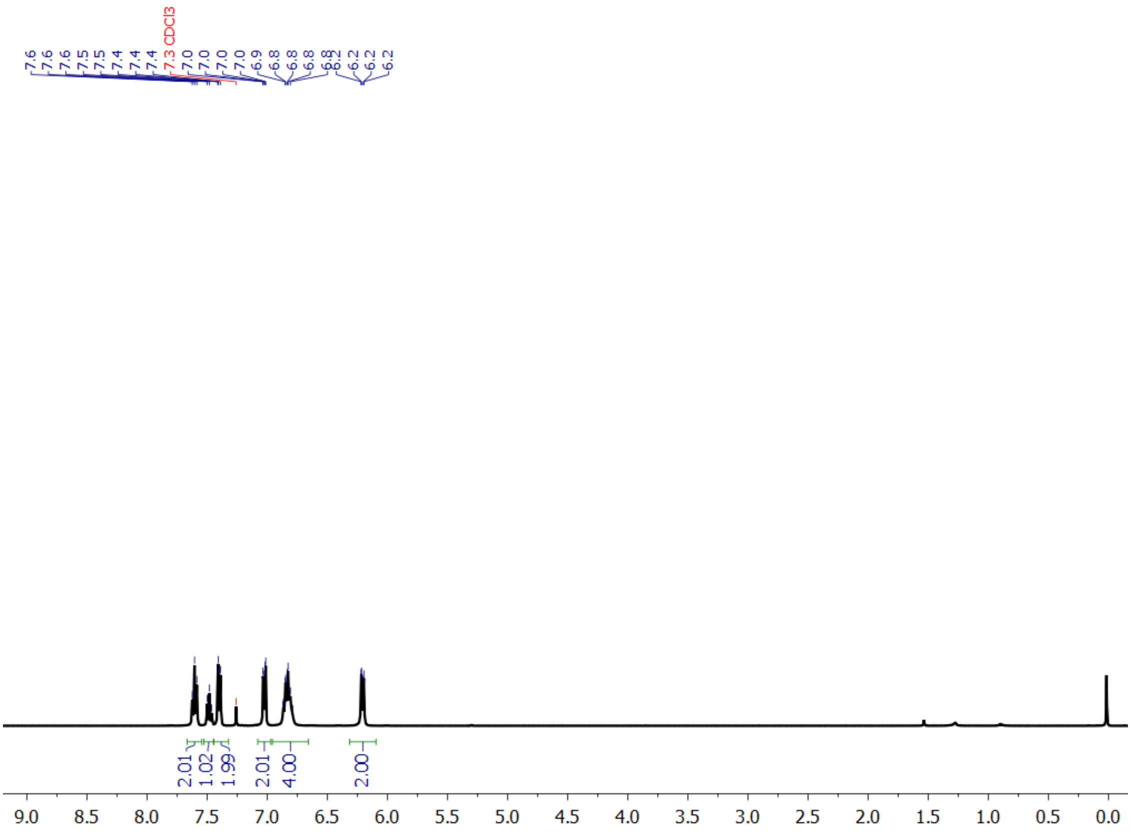

Figure S37: ^{1}H NMR spectrum of compound **1d** (400 MHz, $CDCl_3$, 25 °C).

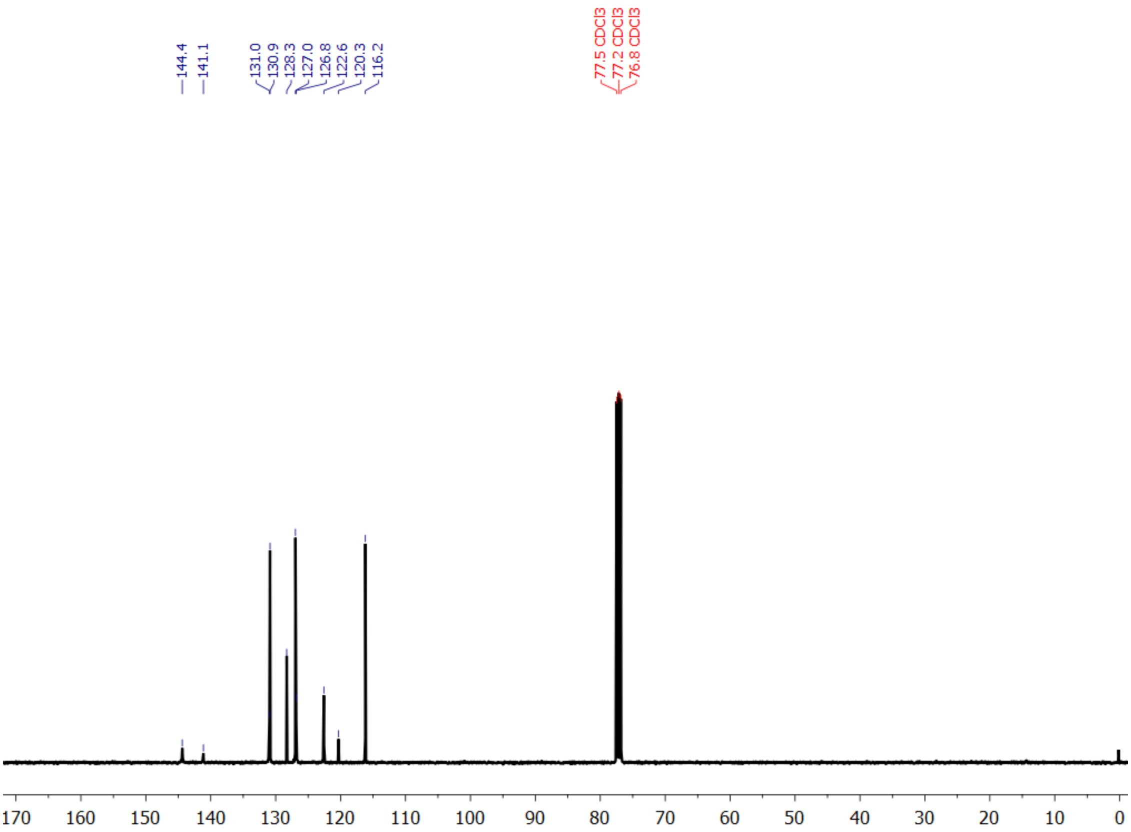

Figure S38: ^{13}C NMR spectrum of compound **1d** (100 MHz, $CDCl_3$, 25 °C).

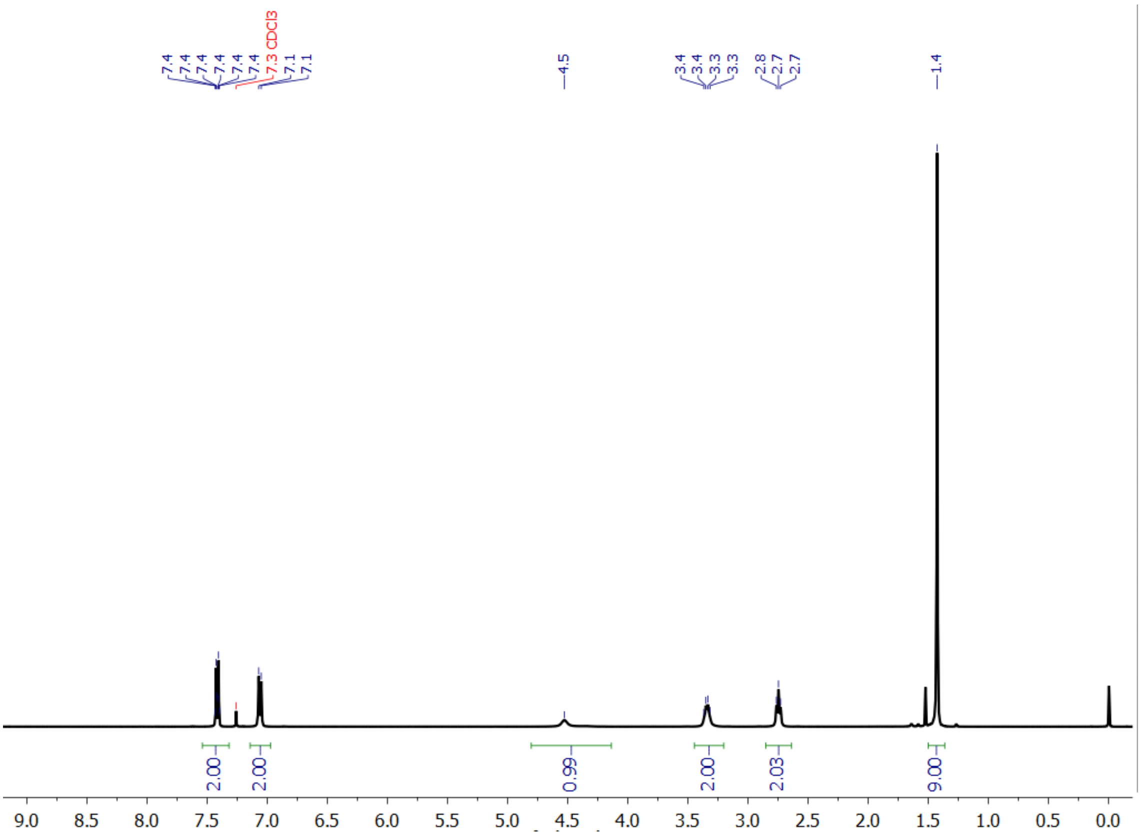

Figure S39: ^{1}H NMR spectrum of compound **A1** (400 MHz, $CDCl_3$, 25 °C).

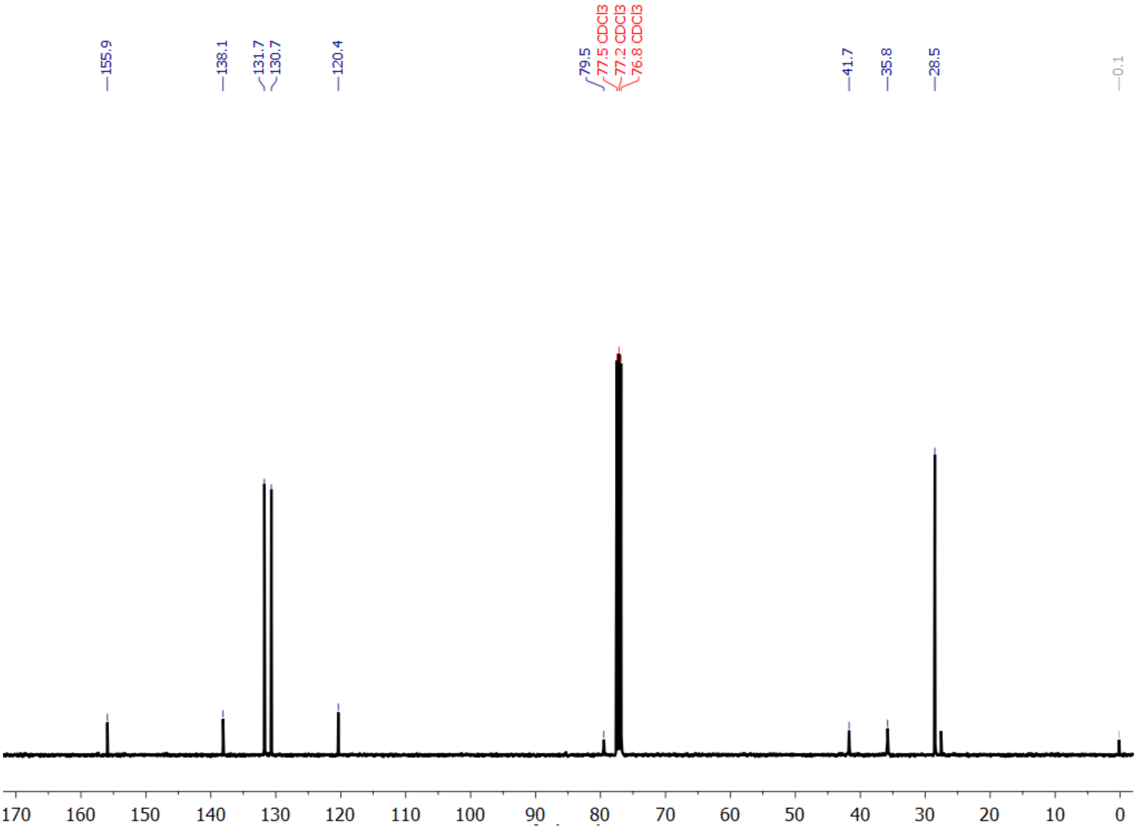

Figure S40: ^{13}C NMR spectrum of compound **A1** (100 MHz, $CDCl_3$, 25 °C).

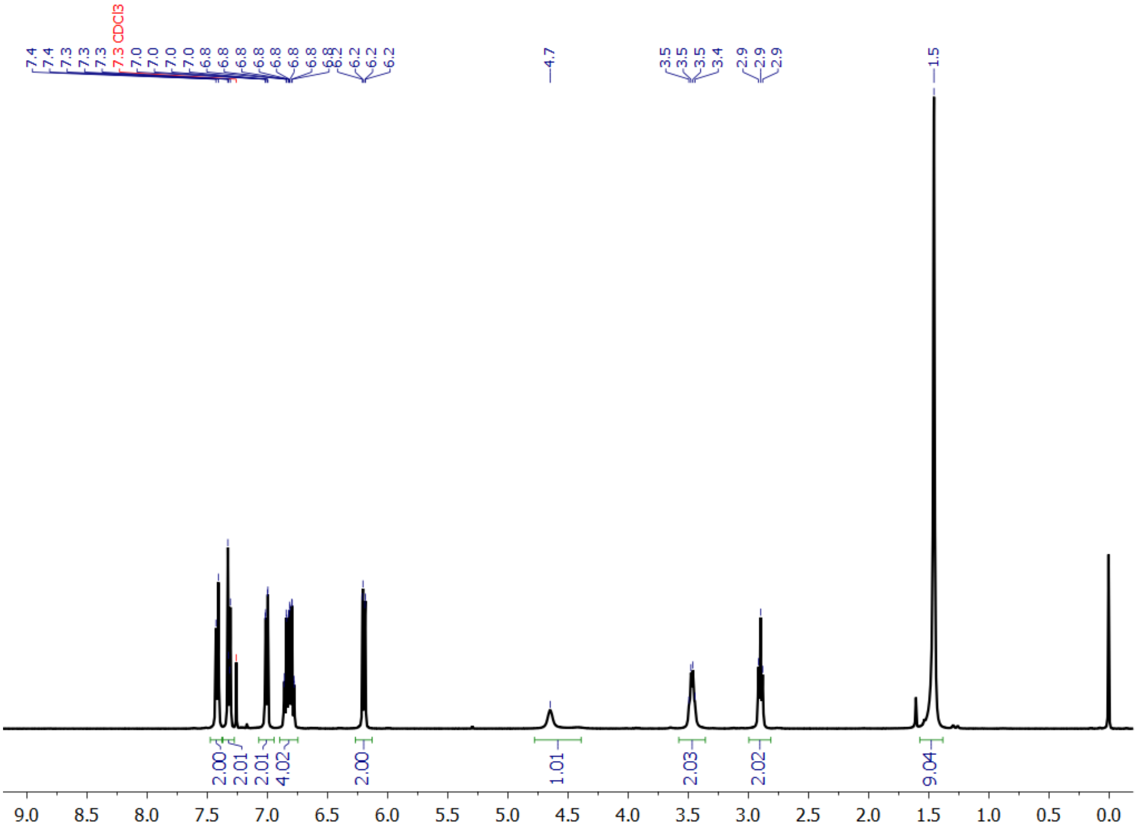

Figure S41: ^{1}H NMR spectrum of compound **A2** (400 MHz, $CDCl_3$, 25 °C).

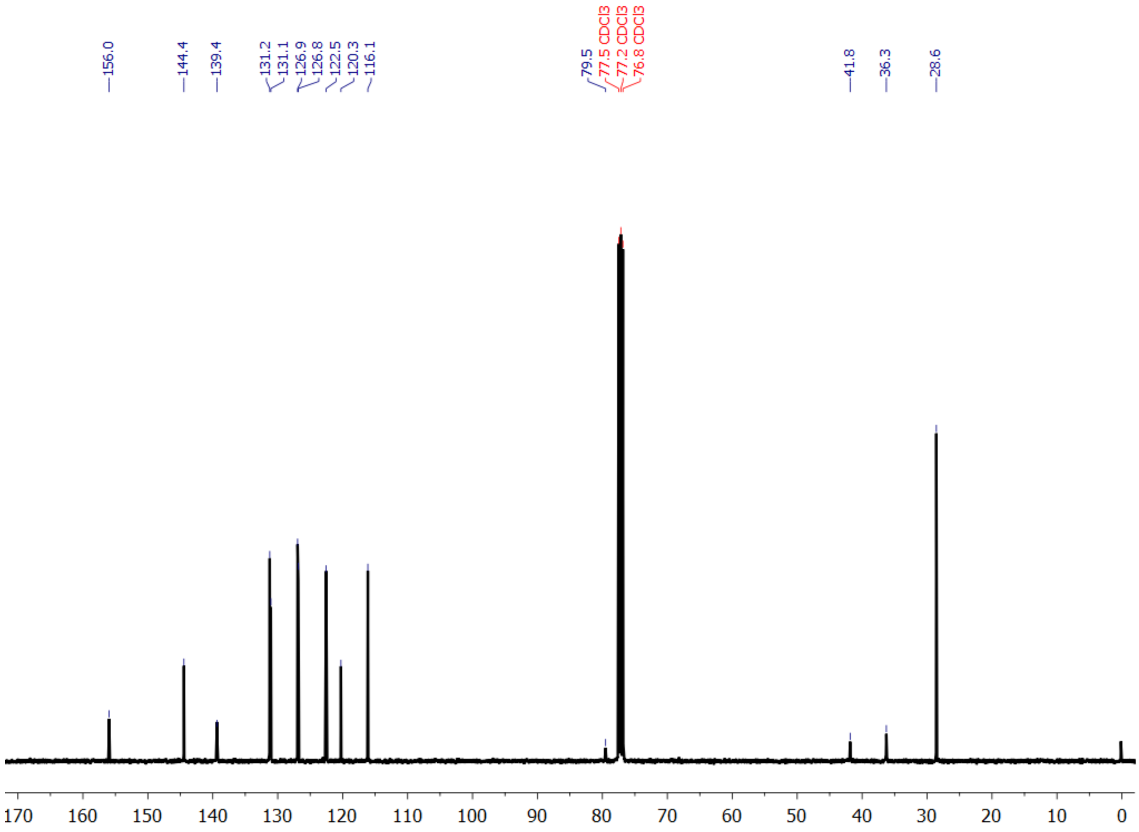

Figure S42: ^{13}C NMR spectrum of compound **A2** (100 MHz, $CDCl_3$, 25 °C).

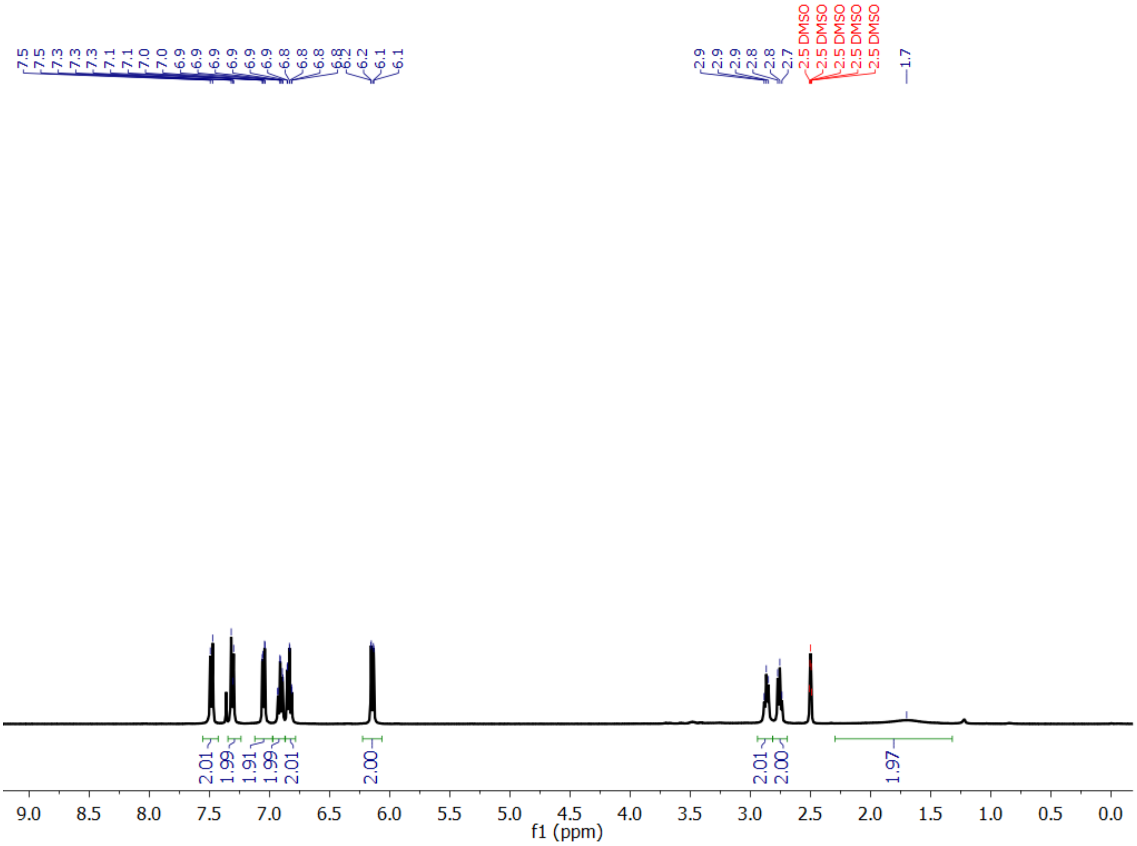

Figure S43: ^{1}H NMR spectrum of compound **A3** (400 MHz, DMSO-d_6, 25 °C).

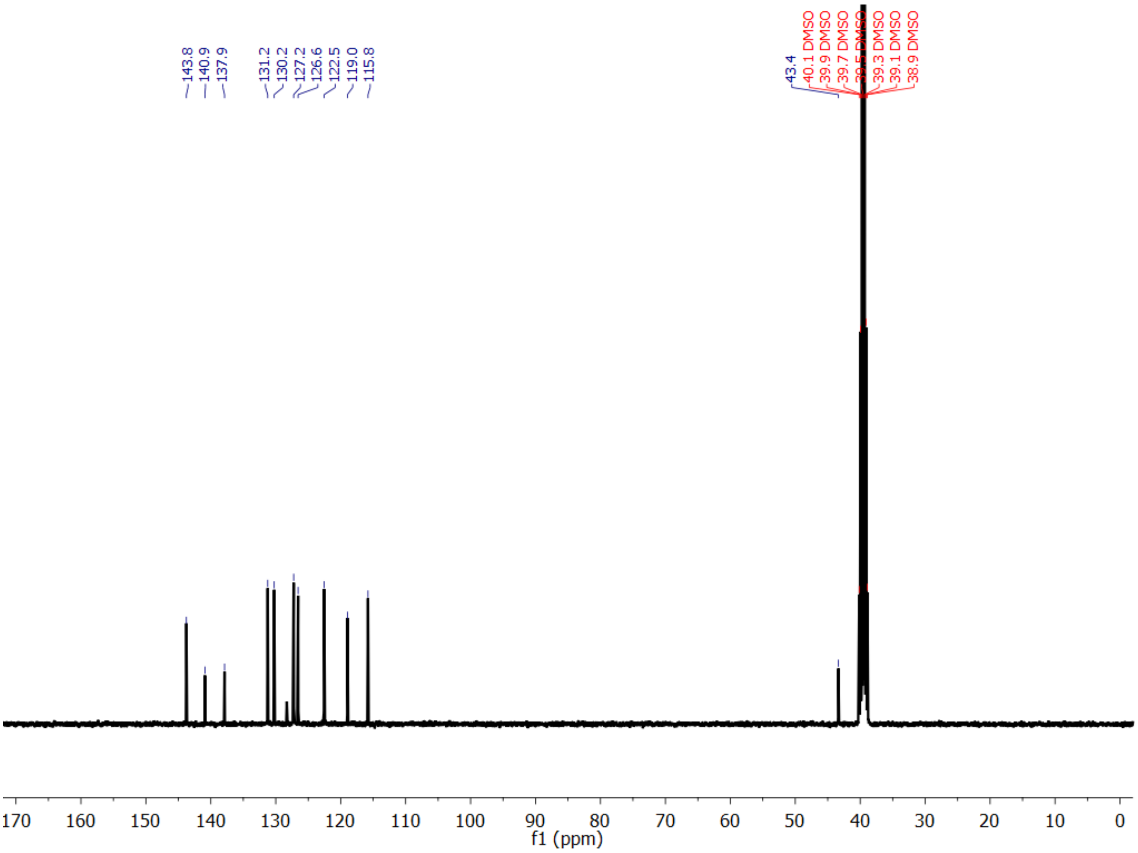

Figure S44: ^{13}C NMR spectrum of compound **A3** (100 MHz, DMSO-d_6, 25 °C).

6. References

(1) Du, Y.-D.; Chen, B.-H.; Shu, W. Direct Access to Primary Amines from Alkenes by Selective Metal-Free Hydroamination. *Angew. Chem. Int. Ed.* **2021**, *60* (18), 9875–9880. https://doi.org/https://doi.org/10.1002/anie.202016679.

(2) Wang, B.; Xiong, D.-C.; Ye, X.-S. Direct C–H Trifluoromethylation of Glycals by Photoredox Catalysis. *Org. Lett.* **2015**, *17* (22), 5698–5701. https://doi.org/10.1021/acs.orglett.5b03016.

(3) Li, L.; Mu, X.; Liu, W.; Wang, Y.; Mi, Z.; Li, C.-J. Simple and Clean Photoinduced Aromatic Trifluoromethylation Reaction. *J. Am. Chem. Soc.* **2016**, *138* (18), 5809–5812. https://doi.org/10.1021/jacs.6b02782.

(4) Natte, K.; Jagadeesh, R. V; He, L.; Rabeah, J.; Chen, J.; Taeschler, C.; Ellinger, S.; Zaragoza, F.; Neumann, H.; Brückner, A.; Beller, M. Palladium-Catalyzed Trifluoromethylation of (Hetero)Arenes with CF3Br. *Angew. Chem. Int. Ed.* **2016**, *55* (8), 2782–2786. https://doi.org/https://doi.org/10.1002/anie.201511131.

(5) Seo, S.; Taylor, J. B.; Greaney, M. F. Silver-Catalysed Trifluoromethylation of Arenes at Room Temperature. *Chem. Commun.* **2013**, *49* (57), 6385–6387. https://doi.org/10.1039/C3CC41829D.

(6) Häring, M.; Abramov, A.; Okumura, K.; Ghosh, I.; König, B.; Yanai, N.; Kimizuka, N.; Díaz Díaz, D. Air-Sensitive Photoredox Catalysis Performed under Aerobic Conditions in Gel Networks. *J. Org. Chem.* **2018**, *83* (15), 7928–7938. https://doi.org/10.1021/acs.joc.8b00797.

(7) Chen, Q.-Y.; Li, Z.-T. Photoinduced Electron-Transfer Reaction of Difluorodiiodomethane with Azaaromatic Compounds and Enamines. *J. Chem. Soc. Perkin Trans. 1* **1993**, No. 6, 645–648. https://doi.org/10.1039/P19930000645.

(8) Du, Y.; Pearson, R. M.; Lim, C.-H.; Sartor, S. M.; Ryan, M. D.; Yang, H.; Damrauer, N. H.; Miyake, G. M. Strongly Reducing, Visible-Light Organic Photoredox Catalysts as Sustainable Alternatives to Precious Metals. *Chem. Eur. J.* **2017**, *23* (46), 10962–10968. https://doi.org/https://doi.org/10.1002/chem.201702926.

(9) Ghosh, I.; Khamrai, J.; Savateev, A.; Shlapakov, N.; Antonietti, M.; König, B. Organic Semiconductor Photocatalyst Can Bifunctionalize Arenes and Heteroarenes. *Science* **2019**, *365* (6451), 360–366. https://doi.org/10.1126/science.aaw3254.

(10) Wiehn, M. S.; Vinogradova, E. V; Togni, A. Electrophilic Trifluoromethylation of Arenes and N-Heteroarenes Using Hypervalent Iodine Reagents. *J. Fluor. Chem.* **2010**, *131* (9), 951–957. https://doi.org/https://doi.org/10.1016/j.jfluchem.2010.06.020.

(11) Egami, H.; Ito, Y.; Ide, T.; Masuda, S.; Hamashima, Y. Simple Photo-Induced Trifluoromethylation of Aromatic Rings. *Synthesis* **2018**, *50* (15), 2948–2953.

(12) Rodrigo, S.; Um, C.; Mixdorf, J. C.; Gunasekera, D.; Nguyen, H. M.; Luo, L. Alternating Current Electrolysis for Organic Electrosynthesis: Trifluoromethylation of (Hetero)Arenes. *Org. Lett.* **2020**, *22* (17), 6719–6723. https://doi.org/10.1021/acs.orglett.0c01906.

(13) Ye, Y.; Lee, S. H.; Sanford, M. S. Silver-Mediated Trifluoromethylation of Arenes Using TMSCF3. *Org. Lett.* **2011**, *13* (20), 5464–5467. https://doi.org/10.1021/ol202174a.

(14) Wang, Y.-F.; Lonca, G. H.; Chiba, S. PhI(OAc)2-Mediated Radical Trifluoromethylation of Vinyl Azides with Me3SiCF3. *Angew. Chem. Int. Ed.* **2014**, *53* (4), 1067–1071. https://doi.org/https://doi.org/10.1002/anie.201307846.

(15) Shen, W.-G.; Wu, Q.-Y.; Gong, X.-Y.; Ao, G.-Z.; Liu, F. A Facile Method for Hydroxytrifluoromethylation of Alkenes with Langlois Reagent and DMSO. *Green Chem.* **2019**, *21* (11), 2983–2987. https://doi.org/10.1039/C9GC00886A.

(16) Shimizu, R.; Egami, H.; Hamashima, Y.; Sodeoka, M. Copper-Catalyzed Trifluoromethylation of Allylsilanes. *Angew. Chem. Int. Ed.* **2012**, *51* (19), 4577–4580. https://doi.org/https://doi.org/10.1002/anie.201201095.

(17) Nagib, D.A.; MacMillan, D.W.C. Trifluoromethylation of arenes and heteroarenes by means of photoredox catalysis. *Nature* **2011**, *480*, 224–228. https://doi:10.1038/nature10647.

(18) Muralirajan, K.; Kancherla, R.; Bau, J.A.; Taksande, M.R.; Qureshi, M; Takanabe, K.; Rueping, M. Exploring the Structure and Performance of Cd−Chalcogenide Photocatalysts in Selective Trifluoromethylation. ACS Catal. **2021**, *11*, 14772−14780. //doi.org/10.1021/acscatal.1c04053.
